# Supplementary material for: Metabolomics Analyses of Cotyledon and Plumule Showing the Potential Domestic Selection in Lotus Breeding
Source: Molecules. 2021 Feb 9;26(4):913. doi: 10.3390/molecules26040913 (PMC7915064; doi:10.3390/molecules26040913)
Supplement: Supplementary file 1 [file molecules-26-00913-s001.zip › molecules-1089745-supplementary/supplementary materials/molecules-1089745-supplementary materials-Figures S1-S4.pdf]

Metabolomics analyses of cotyledon and plumule showing the potential domestic selection in lotus breeding

Huanhuan Qi, Feng Yu, Rebecca Njeri Damaris, Pingfang Yang*

State Key Laboratory of Biocatalysis and Enzyme Engineering, School of Life Sciences, Hubei University, Wuhan, 430062 China

* Correspondence: Prof. Pingfang Yang, e-mail: yangpf@hubu.edu.cn; Tel., 86-27-88663882

	CALS-1	CALS-2	CALS-3	JXLS-1	JXLS-2	JXLS-3	CALP-1	CALP-2	CALP-3	JXLP-1	JXLP-2	JXLP-3	QC01	QC02	QC03
CACL-1	1.00	0.97	0.88	0.86	0.90	0.90	0.37	0.39	0.36	0.39	0.42	0.43	0.53	0.52	0.49
CACL-2	0.97	1.00	0.87	0.89	0.92	0.92	0.36	0.39	0.36	0.40	0.43	0.43	0.48	0.46	0.45
CACL-3	0.88	0.87	1.00	0.81	0.84	0.81	0.34	0.36	0.31	0.35	0.39	0.38	0.50	0.48	0.46
JXCL-1	0.86	0.89	0.81	1.00	0.99	0.98	0.31	0.33	0.29	0.38	0.41	0.41	0.47	0.46	0.45
JXCL-2	0.90	0.92	0.84	0.99	1.00	0.99	0.31	0.33	0.29	0.37	0.41	0.41	0.48	0.46	0.45
JXCL-3	0.90	0.92	0.81	0.98	0.99	1.00	0.31	0.34	0.31	0.38	0.41	0.41	0.46	0.44	0.43
CALP-1	0.37	0.36	0.34	0.31	0.31	0.31	1.00	1.00	0.99	0.92	0.91	0.93	0.91	0.92	0.93
CALP-2	0.39	0.39	0.36	0.33	0.33	0.34	1.00	1.00	0.99	0.92	0.92	0.93	0.91	0.92	0.92
CALP-3	0.36	0.36	0.31	0.29	0.29	0.31	0.99	0.99	1.00	0.92	0.91	0.92	0.89	0.89	0.90
JXLP-1	0.39	0.40	0.35	0.38	0.37	0.38	0.92	0.92	0.92	1.00	0.99	0.99	0.89	0.90	0.91
JXLP-2	0.42	0.43	0.39	0.41	0.41	0.41	0.91	0.92	0.91	0.99	1.00	0.99	0.89	0.90	0.91
JXLP-3	0.43	0.43	0.38	0.41	0.41	0.41	0.93	0.93	0.92	0.99	0.99	1.00	0.90	0.92	0.92
QC01	0.53	0.48	0.50	0.47	0.48	0.46	0.91	0.91	0.89	0.89	0.89	0.90	1.00	1.00	0.99
QC02	0.52	0.46	0.48	0.46	0.46	0.44	0.92	0.92	0.89	0.90	0.90	0.92	1.00	1.00	1.00
QC03	0.49	0.45	0.46	0.45	0.45	0.43	0.93	0.92	0.90	0.91	0.91	0.92	0.99	1.00	1.00

Figure S1. The correlation coefficient of all samples including QC based on identified metabolites. QC, quality control that mixed all of the CL and LP as one sample. CA, China Antique; "JX", JianXuan-17; CL, cotyledon; LP, lotus plumule.

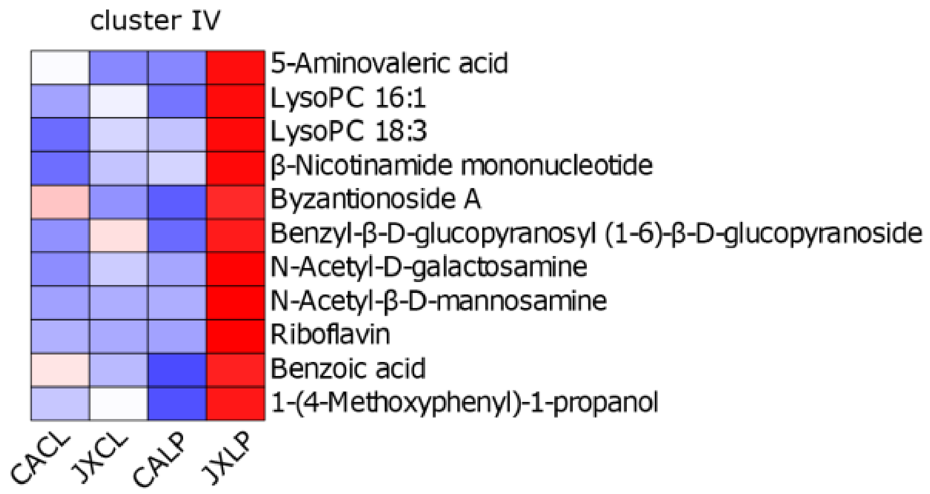

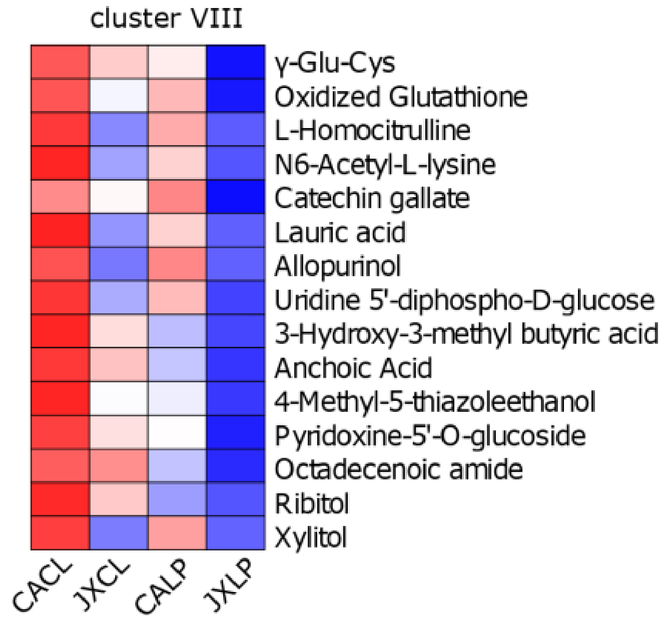

Figure S2: Classification of the metabolites in cluster IV and cluster VIII in the Figure2. "CA", China Antique; "JX", JianXuan-17; CL, cotyledon; LP; lotus plumule

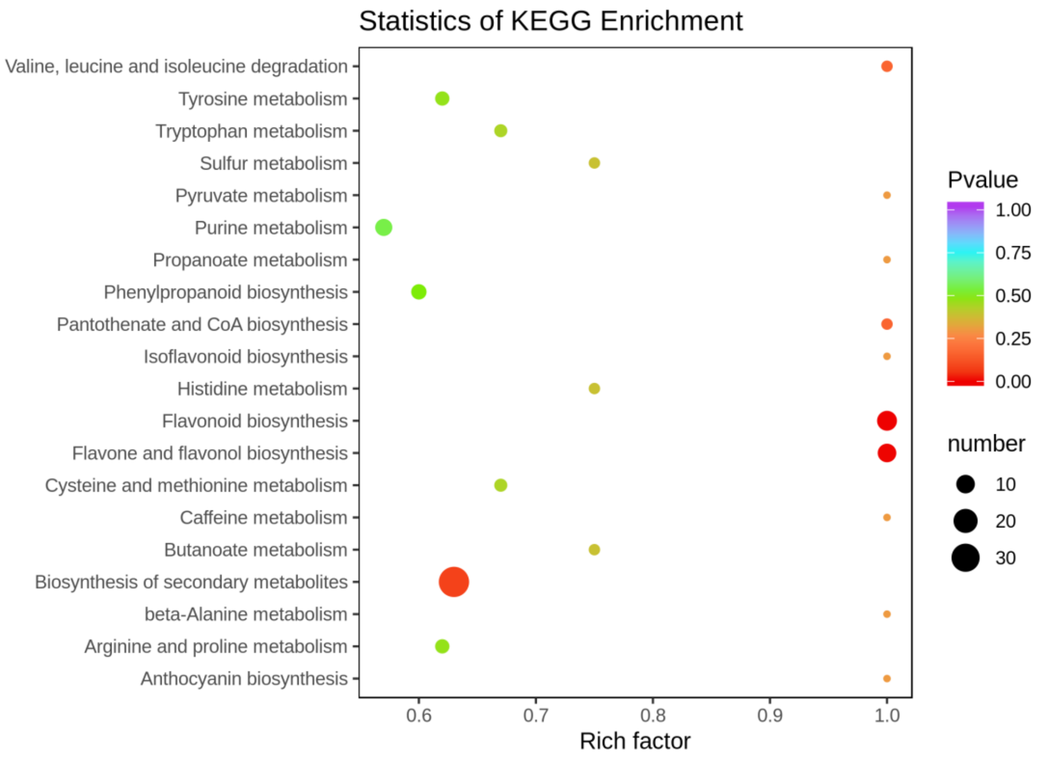

Figure S3: The bubble diagram showing the KEGG pathway enriched for differentially accumulated metabolites in "CA" comparing LP with CL. "CA", China Antique; CL, cotyledon; LP; lotus plumule.

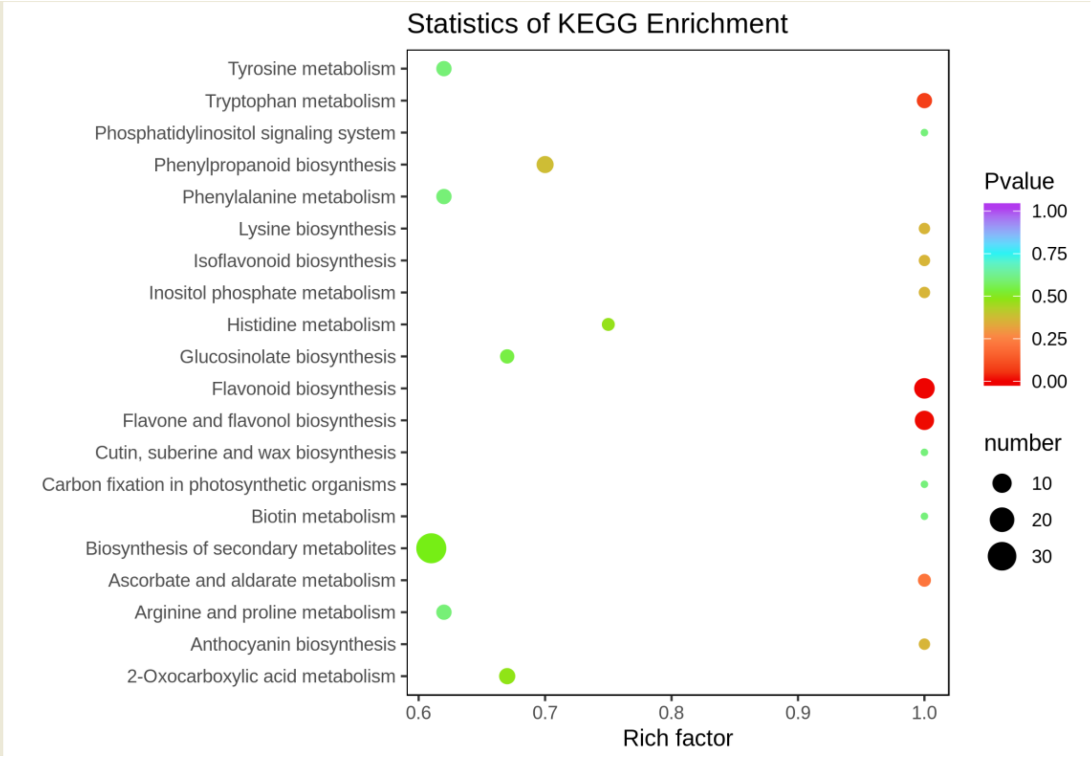

Figure S4: The bubble diagram showing the KEGG pathway enriched for differentially accumulated metabolites in "JX" comparing LP with CL. "JX", JianXuan-17; CL, cotyledon; LP; lotus plumule.
