# Supplementary material for: Metabolomics Analyses of Cotyledon and Plumule Showing the Potential Domestic Selection in Lotus Breeding
Source: Molecules. 2021 Feb 9;26(4):913. doi: 10.3390/molecules26040913 (PMC7915064; doi:10.3390/molecules26040913)
Supplement: Supplementary file 1 [file molecules-26-00913-s001.zip › molecules-1089745-supplementary/supplementary materials/molecules-1089745-table S1.pdf]

Table S1. The detailed information for detected compounds using HPLC-LC-MS/MS.

Compounds	Q1 (Da)	Q3 (Da)	Molecular Weight (Da)	Formula	Ionization model	Class I	Class II	CACL	JXCL	CALP	JXLP	cpd_ID	kegg_map	cluster
Choline	1.04E+02	6.01E+01	1.03E+02	C5H13NO	[M+H]+	Alkaloids	Alkaloids	4.51E+05	4.51E+05	4.61E+05	4.35E+05	C00114	ko00260,ko00564,ko01100,ko02010	cluster1
Indole	1.18E+02	9.10E+01	1.17E+02	C8H7N	[M+H]+	Alkaloids	Plumerane	1.06E+04	9.00E+00	1.24E+04	9.00E+00	C00463	ko00380,ko00400,ko00402,ko01100,ko01110	cluster1
Caaverine	2.68E+02	1.91E+02	2.67E+02	C17H17NO2	[M+H]+	Alkaloids	Alkaloids	2.75E+04	9.00E+00	1.65E+05	5.02E+04	C09368	--	cluster1
Lirinidine	2.82E+02	2.51E+02	2.81E+02	C18H19NO2	[M+H]+	Alkaloids	Alkaloids	2.90E+05	1.41E+05	1.67E+06	6.05E+05	--	--	cluster1
Isoquinoline	1.30E+02	1.03E+02	1.29E+02	C9H7N	[M+H]+	Alkaloids	Isoquinoline alkaloids	2.23E+04	1.23E+04	3.37E+04	3.42E+04	C06323	--	cluster3
N-Acetylputrescine	1.31E+02	1.14E+02	1.30E+02	C6H14N2O	[M+H]+	Alkaloids	Phenolamine	9.00E+00	2.32E+03	1.90E+04	2.82E+04	C02714	ko00330,ko01100	cluster3
Agmatine	1.31E+02	1.14E+02	1.30E+02	C5H14N4	[M+H]+	Alkaloids	Phenolamine	1.55E+03	9.00E+00	2.21E+04	2.60E+04	C00179	ko00330,ko01100	cluster3
Aminopurine	1.36E+02	1.19E+02	1.35E+02	C5H5N5	[M+H]+	Alkaloids	Alkaloids	1.78E+05	1.00E+05	6.37E+05	6.93E+05	--	--	cluster3
Dopamine	1.54E+02	1.37E+02	1.53E+02	C8H11NO2	[M+H]+	Alkaloids	Alkaloids	6.42E+03	9.67E+03	2.42E+06	2.74E+06	--	--	cluster3
Dopamine hydrochloride	1.54E+02	1.37E+02	1.53E+02	C8H11NO2	[M+H]+	Alkaloids	Alkaloids	3.46E+05	1.90E+05	3.12E+07	3.80E+07	--	--	cluster3
Tryptamine	1.61E+02	1.44E+02	1.60E+02	C10H12N2	[M+H]+	Alkaloids	Plumerane	5.58E+04	3.50E+04	1.60E+07	1.00E+07	C00398	ko00380,ko00901,ko01100,ko01110	cluster3
Cotinine	1.77E+02	1.18E+02	1.76E+02	C10H12N2O	[M+H]+	Alkaloids	Alkaloids	9.00E+00	9.00E+00	3.25E+04	2.54E+04	--	--	cluster3
Serotonin	1.77E+02	1.60E+02	1.76E+02	C10H12N2O	[M+H]+	Alkaloids	Plumerane	9.62E+04	6.78E+04	3.00E+07	3.90E+07	C00780	ko00380,ko01100	cluster3
Higenamine	2.72E+02	2.55E+02	2.71E+02	C16H17NO3	[M+H]+	Alkaloids	Isoquinoline alkaloids	9.00E+00	9.00E+00	3.86E+06	2.35E+06	C06346	--	cluster3
Roemerine	2.80E+02	2.49E+02	2.79E+02	C18H17NO2	[M+H]+	Alkaloids	Alkaloids	4.52E+04	4.73E+04	2.78E+05	3.00E+05	--	--	cluster3
Nornuciferine	2.82E+02	2.65E+02	2.81E+02	C18H19NO2	[M+H]+	Alkaloids	Aporphine alkaloids	3.84E+04	1.14E+05	1.10E+06	2.57E+06	--	--	cluster3
Floribundine	2.82E+02	2.51E+02	2.81E+02	C18H19NO2	[M+H]+	Alkaloids	Alkaloids	2.85E+05	1.37E+05	1.14E+06	6.54E+05	--	--	cluster3
p-Coumaroyltyramine	2.84E+02	1.47E+02	2.83E+02	C17H17NO3	[M+H]+	Alkaloids	Phenolamine	9.00E+00	9.00E+00	1.54E+04	1.04E+04	--	--	cluster3
Nuciferine	2.96E+02	2.50E+02	2.95E+02	C19H21NO2	[M+H]+	Alkaloids	Aporphine alkaloids	6.81E+05	1.30E+06	2.76E+07	4.61E+07	--	--	cluster3
Methylcoclaurine	3.00E+02	2.69E+02	2.99E+02	C18H21NO3	[M+H]+	Alkaloids	Alkaloids	3.12E+06	3.72E+06	4.52E+07	5.88E+07	C05176	ko00950,ko01100,ko01110	cluster3
Norarmepavine	3.00E+02	2.83E+02	2.99E+02	C18H21NO3	[M+H]+	Alkaloids	Quinoline alkaloids	9.00E+00	9.00E+00	2.46E+05	2.37E+05	--	--	cluster3
Lotusine	3.14E+02	2.99E+02	3.13E+02	C19H23NO3	[M+H]+	Alkaloids	Isoquinoline alkaloids	2.34E+05	2.35E+05	1.49E+06	1.39E+06	C17567	--	cluster3
Armepavine	3.14E+02	2.83E+02	3.13E+02	C19H23NO3	[M+H]+	Alkaloids	Isoquinoline alkaloids	4.93E+06	3.26E+06	6.19E+07	4.41E+07	C09342	--	cluster3
Liensinine	6.11E+02	2.06E+02	6.10E+02	C37H42N2O6	[M+H]+	Alkaloids	Alkaloids	3.00E+05	8.00E+05	5.75E+07	3.21E+07	--	--	cluster3
5-Aminocycloheptane-1,2,3-triol	1.62E+02	7.91E+01	1.61E+02	C7H15NO3	[M+H]+	Alkaloids	Alkaloids	1.03E+04	1.00E+04	7.31E+03	1.07E+04	--	--	cluster5
Trigonelline	1.38E+02	9.41E+01	1.37E+02	C7H7NO2	[M+H]+	Alkaloids	Alkaloids	5.27E+07	3.83E+07	2.17E+07	3.05E+07	C01004	ko00760	cluster6
Indole-3-carboxylic acid	1.60E+02	1.16E+02	1.61E+02	C9H7NO2	[M-H]-	Alkaloids	Plumerane	8.96E+05	7.79E+05	1.28E+05	2.82E+05	--	--	cluster6
Indole-5-carboxylic acid	1.60E+02	1.16E+02	1.61E+02	C9H7NO2	[M-H]-	Alkaloids	Plumerane	8.53E+05	7.38E+05	1.23E+05	2.55E+05	--	--	cluster6
10-Formyl-THF	4.74E+02	3.27E+02	4.73E+02	C20H23N7O7	[M+H]+	Alkaloids	Alkaloids	3.74E+07	5.12E+07	1.95E+06	2.41E+06	C00234	ko00670,ko00970,ko01100,ko01200	cluster6
Betaine	1.18E+02	5.90E+01	1.17E+02	C5H11NO2	[M+H]+	Alkaloids	Alkaloids	2.45E+06	6.02E+05	1.01E+06	7.85E+05	C00719	ko00260,ko01100,ko02010	cluster9
N-Benzylmethylene isomethylamine	1.20E+02	1.03E+02	1.19E+02	C8H9N	[M+H]+	Alkaloids	Alkaloids	1.69E+07	6.29E+06	2.54E+07	1.54E+07	--	--	cluster10
4,5,6-Trihydroxy-2-cyclohexen-1-ylideneacetonitrile	1.68E+02	1.05E+02	1.67E+02	C8H9NO3	[M+H]+	Alkaloids	Alkaloids	4.78E+05	1.53E+05	8.21E+05	4.41E+05	--	--	cluster10
N,N-Dimethylglycine	1.04E+02	8.60E+01	1.03E+02	C4H9NO2	[M+H]+	Amino acids and derivatives	Amino acids and derivatives	1.09E+06	9.64E+05	1.28E+06	8.93E+05	C01026	ko00260,ko01100	cluster1
D-Serine	1.04E+02	7.40E+01	1.05E+02	C3H7NO3	[M-H]-	Amino acids and derivatives	Amino acids and derivatives	3.69E+04	2.11E+04	7.20E+04	1.57E+04	C00740	ko00260,ko01100,ko01502	cluster1
Pipecolic acid	1.30E+02	8.41E+01	1.29E+02	C6H11NO2	[M+H]+	Amino acids and derivatives	Amino acids and derivatives	1.90E+06	1.29E+06	1.33E+07	3.14E+06	--	--	cluster1
L-Methionine	1.50E+02	6.10E+01	1.49E+02	C5H11NO2S	[M+H]+	Amino acids and derivatives	Amino acids and derivatives	2.97E+06	1.50E+06	6.67E+06	2.11E+06	C00073	ko00270,ko00966,ko00970,ko01100,ko01110,ko01210,ko01230	cluster1
Methionine methyl ester	1.64E+02	1.04E+02	1.63E+02	C6H13NO2S	[M+H]+	Amino acids and derivatives	Amino acids and derivatives	1.02E+04	4.43E+03	2.55E+04	9.41E+03	--	--	cluster1
N-Glycyl-L-leucine	1.89E+02	8.60E+01	1.88E+02	C8H16N2O3	[M+H]+	Amino acids and derivatives	Amino acids and derivatives	6.69E+04	3.87E+04	1.97E+05	8.64E+04	C02155	--	cluster1
Glycylisoleucine	1.89E+02	8.61E+01	1.88E+02	C8H16N2O3	[M+H]+	Amino acids and derivatives	Amino acids and derivatives	5.05E+04	2.98E+04	1.54E+05	6.55E+04	--	--	cluster1
Glycylphenylalanine	2.23E+02	1.20E+02	2.22E+02	C11H14N2O3	[M+H]+	Amino acids and derivatives	Amino acids and derivatives	5.36E+04	2.46E+04	3.72E+05	1.13E+05	--	--	cluster1
DL-Alanyl-DL-phenylalanine	2.37E+02	1.20E+02	2.36E+02	C12H16N2O3	[M+H]+	Amino acids and derivatives	Amino acids and derivatives	1.56E+04	9.00E+00	1.23E+05	3.52E+04	--	--	cluster1
D-Alanyl-D-Alanine	1.59E+02	8.80E+01	1.60E+02	C6H12N2O3	[M-H]-	Amino acids and derivatives	Amino acids and derivatives	9.00E+00	4.54E+03	6.13E+03	5.07E+03	C00993	ko01100,ko01502	cluster2
L-Homocystine	2.69E+02	1.36E+02	2.68E+02	C8H16N2O4S2	[M+H]+	Amino acids and derivatives	Amino acids and derivatives	2.35E+04	4.22E+04	3.63E+04	4.34E+04	C01817	ko00270	cluster2
Histamine	1.12E+02	9.50E+01	1.11E+02	C5H9N3	[M+H]+	Amino acids and derivatives	Amino acids and derivatives	1.31E+04	5.61E+03	1.09E+05	7.84E+04	C00388	ko00340,ko01100,ko01110	cluster3
L-Proline	1.16E+02	7.01E+01	1.15E+02	C5H9NO2	[M+H]+	Amino acids and derivatives	Amino acids and derivatives	9.64E+05	7.19E+05	2.34E+06	1.31E+06	C00148	ko00330,ko00332,ko00970,ko01100,ko01110,ko01230,ko02010	cluster3
L-Valine	1.18E+02	7.21E+01	1.17E+02	C5H11NO2	[M+H]+	Amino acids and derivatives	Amino acids and derivatives	6.81E+06	2.27E+06	1.42E+07	7.83E+06	C00183	ko00280,ko00290,ko00460,ko00770,ko00966,ko00970,ko01100,ko01110,ko01210,ko01230,ko02010	cluster3

L-Leucine	1.32E+02	8.62E+01	1.31E+02	C6H13NO2	[M+H]+	Amino acids and derivatives	Amino acids and derivatives	6.58E+05	2.18E+05	1.82E+06	9.51E+05	C00123	ko00280,ko00290,ko00966,ko00970,ko01100,ko01110,ko01210,ko01230,ko02010	cluster3
L-Asparagine	1.33E+02	7.40E+01	1.32E+02	C4H8N2O3	[M+H]+	Amino acids and derivatives	Amino acids and derivatives	1.40E+06	1.42E+06	2.68E+06	2.01E+06	C00152	ko00250,ko00460,ko00970,ko01100,ko01110,ko01230	cluster3
DL-Homocysteine	1.36E+02	1.18E+02	1.35E+02	C4H9NO2S	[M+H]+	Amino acids and derivatives	Amino acids and derivatives	9.00E+00	3.23E+03	9.31E+03	7.42E+03	C00155	ko00270,ko00920,ko01100,ko01110,ko01230	cluster3
L-Tyramine	1.38E+02	1.03E+02	1.37E+02	C8H11NO	[M+H]+	Amino acids and derivatives	Amino acids and derivatives	8.12E+05	6.36E+05	9.51E+06	1.01E+07	C00483	ko00350,ko00950,ko01100,ko01110	cluster3
L-Glutamine	1.47E+02	8.40E+01	1.46E+02	C5H10N2O3	[M+H]+	Amino acids and derivatives	Amino acids and derivatives	1.17E+07	5.75E+06	3.50E+07	1.92E+07	C00064	ko00220,ko00230,ko00240,ko00250,ko00630,ko00910,ko00970,ko01100,ko01230,ko02010	cluster3
L-(+)-Lysine	1.47E+02	8.40E+01	1.46E+02	C6H14N2O2	[M+H]+	Amino acids and derivatives	Amino acids and derivatives	1.18E+07	5.53E+06	3.59E+07	1.95E+07	C00047	ko00300,ko00310,ko00780,ko00960,ko00970,ko01100,ko01110,ko01210,ko01230,ko02010	cluster3
O-Acetylserine	1.48E+02	8.80E+01	1.47E+02	C5H9NO4	[M+H]+	Amino acids and derivatives	Amino acids and derivatives	2.25E+05	3.02E+05	4.69E+05	5.54E+05	C00979	ko00270,ko00908,ko00920,ko00998,ko01100,ko01110,ko01200,ko01230,ko04122	cluster3
L-Histidine	1.56E+02	1.10E+02	1.55E+02	C6H9N3O2	[M+H]+	Amino acids and derivatives	Amino acids and derivatives	4.44E+06	1.32E+06	1.90E+07	1.44E+07	C00135	ko00340,ko00410,ko00970,ko01100,ko01110,ko01230,ko02010	cluster3
Methionine sulfoxide	1.66E+02	1.02E+02	1.65E+02	C5H11NO3S	[M+H]+	Amino acids and derivatives	Amino acids and derivatives	4.69E+06	2.25E+06	6.05E+06	6.35E+06	--	--	cluster3
1-Methylhistidine	1.70E+02	1.24E+02	1.69E+02	C7H11N3O2	[M+H]+	Amino acids and derivatives	Amino acids and derivatives	3.37E+05	3.42E+05	1.95E+06	1.84E+06	C01152	ko00340,ko01100	cluster3
N-Acetyl-L-leucine	1.74E+02	8.60E+01	1.73E+02	C8H15NO3	[M+H]+	Amino acids and derivatives	Amino acids and derivatives	1.00E+05	4.38E+04	1.88E+05	1.36E+05	C02710	--	cluster3
H-HomoArg-OH	1.89E+02	1.44E+02	1.88E+02	C7H16N4O2	[M+H]+	Amino acids and derivatives	Amino acids and derivatives	1.95E+05	9.88E+04	4.23E+05	4.40E+05	C01924	--	cluster3
2,6-Diaminooimelic acid	1.91E+02	1.28E+02	1.90E+02	C7H14N2O4	[M+H]+	Amino acids and derivatives	Amino acids and derivatives	5.28E+04	1.09E+04	1.30E+05	1.03E+05	C00666	ko00300,ko01100,ko01110,ko01230	cluster3
3,4-Dihydroxy-DL-phenylalanine	1.98E+02	1.52E+02	1.97E+02	C9H11NO4	[M+H]+	Amino acids and derivatives	Amino acids and derivatives	1.40E+04	8.59E+03	2.18E+04	1.52E+04	C00355	ko00350,ko00950,ko00965,ko01100,ko01110	cluster3
N-α-Acetyl-L-arginine	2.17E+02	1.58E+02	2.16E+02	C8H16N4O3	[M+H]+	Amino acids and derivatives	Amino acids and derivatives	9.00E+00	9.00E+00	7.71E+03	2.32E+04	--	--	cluster3
S-(5'-Adenosy)-L-homocysteine	3.85E+02	2.50E+02	3.84E+02	C14H20N6O5S	[M+H]+	Amino acids and derivatives	Amino acids and derivatives	1.69E+05	1.13E+05	1.58E+06	1.32E+06	C00021	ko00270,ko01100,ko01230	cluster3
5-Aminovaleric acid	1.18E+02	1.01E+02	1.17E+02	C5H11NO2	[M+H]+	Amino acids and derivatives	Amino acids and derivatives	6.89E+03	5.98E+03	5.97E+03	8.82E+03	C00431	ko00310,ko00330,ko01100	cluster4
N-Acetylglycine	1.16E+02	7.40E+01	1.17E+02	C4H7NO3	[M-H]-	Amino acids and derivatives	Amino acids and derivatives	2.13E+04	3.41E+04	6.27E+03	1.53E+04	--	--	cluster5
L-PyroglutamicAcid	1.28E+02	8.20E+01	1.29E+02	C5H7NO3	[M-H]-	Amino acids and derivatives	Amino acids and derivatives	2.27E+04	3.00E+04	1.89E+04	2.33E+04	C01879	ko00480,ko01100	cluster5
1,2-N-Methylpipecolic acid	1.44E+02	8.41E+01	1.43E+02	C7H13NO2	[M+H]+	Amino acids and derivatives	Amino acids and derivatives	2.61E+05	4.74E+05	1.68E+05	5.83E+05	--	--	cluster5
S-(methyl)glutathione	3.22E+02	1.30E+02	3.21E+02	C11H19N3O6S	[M+H]+	Amino acids and derivatives	Amino acids and derivatives	4.44E+05	1.00E+06	3.52E+05	4.59E+05	C11347	--	cluster5
L-Glutamic acid	1.48E+02	8.40E+01	1.47E+02	C5H9NO4	[M+H]+	Amino acids and derivatives	Amino acids and derivatives	4.10E+07	4.27E+07	3.08E+07	2.74E+07	C00025	ko00220,ko00250,ko00330,ko00332,ko00340,ko00430,ko00480,ko00630,ko00650,ko00660,ko00860,ko00910,ko00970,ko00998,ko01100,ko01110,ko01200,ko01210,ko01230,ko02010	cluster6
N-Acetylthreonine	1.60E+02	9.80E+01	1.61E+02	C6H11NO4	[M-H]-	Amino acids and derivatives	Amino acids and derivatives	6.85E+04	7.29E+04	3.20E+04	2.87E+04	--	--	cluster6
N-Acetylaspartate	1.74E+02	8.80E+01	1.75E+02	C6H9NO5	[M-H]-	Amino acids and derivatives	Amino acids and derivatives	4.56E+04	4.38E+04	9.00E+00	9.00E+00	C01042	ko00250,ko01100	cluster6
N-α-Acetyl-L-glutamine	1.87E+02	1.25E+02	1.88E+02	C7H12N2O4	[M-H]-	Amino acids and derivatives	Amino acids and derivatives	4.70E+05	5.03E+05	5.39E+04	8.54E+04	--	--	cluster6
L-2-chlorophenylalanine	2.00E+02	1.54E+02	1.99E+02	C9H10ClNO2	[M+H]+	Amino acids and derivatives	Amino acids and derivatives	1.24E+06	1.39E+06	5.68E+05	4.28E+05	--	--	cluster6
Asp-phe	2.81E+02	1.66E+02	2.80E+02	C13H16N2O5	[M+H]+	Amino acids and derivatives	Amino acids and derivatives	2.52E+05	2.23E+05	4.86E+04	6.58E+04	--	--	cluster6
Nicotianamine	3.04E+02	1.85E+02	3.03E+02	C12H21N3O6	[M+H]+	Amino acids and derivatives	Amino acids and derivatives	3.62E+06	5.42E+06	5.46E+05	5.99E+05	--	--	cluster6
2-Aminoisobutyric acid	1.04E+02	5.80E+01	1.03E+02	C4H9NO2	[M+H]+	Amino acids and derivatives	Amino acids and derivatives	9.48E+06	9.62E+06	9.65E+06	9.30E+06	C03665	--	cluster7
L-(-)-Threonine	1.20E+02	7.40E+01	1.19E+02	C4H9NO3	[M+H]+	Amino acids and derivatives	Amino acids and derivatives	2.15E+06	2.37E+06	2.28E+06	1.82E+06	C00188	ko00260,ko00261,ko00290,ko00860,ko00970,ko01100,ko01110,ko01230,ko02010	cluster7
N6-Acetyl-L-lysine	1.89E+02	1.26E+02	1.88E+02	C8H16N2O3	[M+H]+	Amino acids and derivatives	Amino acids and derivatives	7.07E+05	2.40E+05	4.47E+05	1.24E+05	C02727	ko00310,ko01100	cluster8
L-Homocitrulline	1.90E+02	1.27E+02	1.89E+02	C7H15N3O3	[M+H]+	Amino acids and derivatives	Amino acids and derivatives	9.74E+04	2.73E+04	7.21E+04	1.75E+04	C02427	--	cluster8
γ-Glu-Cys	2.49E+02	1.28E+02	2.50E+02	C8H14N2O5S	[M-H]-	Amino acids and derivatives	Amino acids and derivatives	6.96E+04	4.97E+04	4.39E+04	9.00E+00	C00669	ko00480,ko01100	cluster8

Oxidized Glutathione	6.11E+02	3.06E+02	6.12E+02	C20H32N6O12S2	[M-H]-	Amino acids and derivatives	Amino acids and derivatives	1.98E+06	1.33E+06	1.62E+06	5.29E+05	--	--	cluster8
3-Hydroxy-3-methylpentane-1,5-dioic acid	1.61E+02	9.90E+01	1.62E+02	C6H10O5	[M-H]-	Amino acids and derivatives	Amino acids and derivatives	1.96E+06	8.35E+05	7.53E+05	1.10E+06	C03761	--	cluster9
5-Hydroxy-L-tryptophan	2.21E+02	2.04E+02	2.20E+02	C11H12N2O3	[M+H]+	Amino acids and derivatives	Amino acids and derivatives	7.08E+04	2.89E+04	9.00E+00	9.00E+00	C00643	ko00380,ko01100	cluster9
N-Acetyl-L-tyrosine	2.24E+02	1.36E+02	2.23E+02	C11H13NO4	[M+H]+	Amino acids and derivatives	Amino acids and derivatives	1.24E+05	5.39E+04	2.52E+04	5.36E+04	--	--	cluster9
Acetyltryptophan	2.45E+02	2.03E+02	2.46E+02	C13H14N2O3	[M-H]-	Amino acids and derivatives	Amino acids and derivatives	6.06E+05	1.28E+05	8.10E+04	1.86E+04	C03137	--	cluster9
N-Acetyl-DL-tryptophan	2.89E+02	2.03E+02	2.90E+02	C15H18N2O4	[M-H]-	Amino acids and derivatives	Amino acids and derivatives	3.88E+05	8.38E+04	5.35E+04	1.14E+04	--	--	cluster9
L-Phenylalanine	1.66E+02	1.20E+02	1.65E+02	C9H11NO2	[M+H]+	Amino acids and derivatives	Amino acids and derivatives	4.51E+05	1.50E+05	7.39E+05	4.19E+05	C00079	ko00360,ko00400,ko00460,ko00940,ko00960,ko00966,ko00970,ko01100,ko01110,ko01210,ko01230,ko02010	cluster10
L-(+)-Arginine	1.75E+02	1.16E+02	1.74E+02	C6H14N4O2	[M+H]+	Amino acids and derivatives	Amino acids and derivatives	1.91E+07	2.34E+06	1.57E+07	8.45E+06	C00062	ko00220,ko00261,ko00330,ko00472,ko00970,ko00998,ko01100,ko01110,ko01230,ko02010	cluster10
L-Citrulline	1.76E+02	1.13E+02	1.75E+02	C6H13N3O3	[M+H]+	Amino acids and derivatives	Amino acids and derivatives	5.71E+05	2.75E+05	5.47E+05	3.35E+05	C00327	ko00220,ko01100,ko01110,ko01230	cluster10
L-(-)-Tyrosine	1.82E+02	1.36E+02	1.81E+02	C9H11NO3	[M+H]+	Amino acids and derivatives	Amino acids and derivatives	3.83E+07	1.32E+07	2.71E+07	2.58E+07	C00082	ko00130,ko00261,ko00350,ko00360,ko00400,ko00460,ko00730,ko00940,ko00950,ko00965,ko00966,ko00970,ko01100,ko01110,ko01210,ko01230	cluster10
L-Tryptophan	2.03E+02	1.16E+02	2.04E+02	C11H12N2O2	[M-H]-	Amino acids and derivatives	Amino acids and derivatives	5.80E+06	1.42E+06	7.54E+06	4.30E+06	C00078	ko00260,ko00380,ko00400,ko00901,ko00966,ko00970,ko01100,ko01110,ko01210,ko01230	cluster10
Naringenin chalcone	2.73E+02	1.53E+02	2.72E+02	C15H12O5	[M+H]+	Flavonoids	Chalcones	3.38E+03	3.12E+03	3.18E+05	4.65E+04	C06561	ko00941,ko01100,ko01110	cluster1
Butin	2.73E+02	1.53E+02	2.72E+02	C15H12O5	[M+H]+	Flavonoids	Dihydroflavone	3.23E+03	2.80E+03	3.25E+05	4.58E+04	C09614	ko00941	cluster1
Naringenin	2.71E+02	1.51E+02	2.72E+02	C15H12O5	[M-H]-	Flavonoids	Dihydroflavone	4.94E+03	4.63E+03	6.23E+05	8.83E+04	C00509	ko00941,ko00943,ko01100,ko01110	cluster1
Pinobanksin	2.71E+02	1.51E+02	2.72E+02	C15H12O5	[M-H]-	Flavonoids	Dihydroflavonol	6.40E+03	4.14E+03	6.48E+05	9.66E+04	C09826	ko00941	cluster1
5,7,4-trihydroxy-dihydroflavone	2.73E+02	1.53E+02	2.72E+02	C15H12O5	[M+H]+	Flavonoids	Dihydroflavone	3.06E+03	3.77E+03	3.35E+05	4.94E+04	--	--	cluster1
Eriodictyol	2.87E+02	1.35E+02	2.88E+02	C15H12O6	[M-H]-	Flavonoids	Dihydroflavone	9.00E+00	9.00E+00	3.13E+05	7.05E+04	C05631	ko00941,ko01100,ko01110	cluster1
Isorhamnetin	3.15E+02	1.51E+02	3.16E+02	C16H12O7	[M-H]-	Flavonoids	Flavonols	9.00E+00	9.00E+00	1.53E+05	1.66E+04	C10084	--	cluster1
Isorhamnetin-3-O-arabinoside	4.49E+02	3.17E+02	4.48E+02	C21H20O11	[M+H]+	Flavonoids	Flavonols	9.00E+00	9.00E+00	2.79E+05	6.24E+04	--	--	cluster1
Malonylglygenistin	5.19E+02	2.71E+02	5.18E+02	C24H22O13	[M+H]+	Flavonoids	Isoflavones	1.78E+04	2.12E+04	2.47E+05	2.86E+04	--	--	cluster1
Isorhamnetin-O-acetyl-hexoside	5.19E+02	3.14E+02	5.20E+02	C24H24O13	[M-H]-	Flavonoids	Flavonols	9.00E+00	9.00E+00	6.83E+04	6.89E+03	--	--	cluster1
Tricin O-saccharic acid	5.21E+02	3.29E+02	5.22E+02	C23H22O14	[M-H]-	Flavonoids	Flavonoid	8.67E+03	9.00E+00	1.60E+05	3.51E+04	--	--	cluster1
Kaempferol-O-glucoside-O-glucoside	6.11E+02	2.87E+02	6.10E+02	C27H30O16	[M+H]+	Flavonoids	Flavonols	9.00E+00	9.00E+00	2.99E+05	5.71E+04	--	--	cluster1
8-C-Hexosyl-luteolin O-hexoside	6.11E+02	3.17E+02	6.10E+02	C27H30O16	[M+H]+	Flavonoids	Flavonoid carbonoside	9.00E+00	9.00E+00	7.15E+05	1.64E+05	--	--	cluster1
Quercetin-3-O-(2-O-α-L-rhamnopyranosyl)-β-D-galactopyranosic	6.11E+02	3.03E+02	6.10E+02	C27H30O16	[M+H]+	Flavonoids	Flavonoid	9.00E+00	9.00E+00	1.60E+05	2.85E+04	--	--	cluster1
Isorhamnetin-3-O-neohesperidoside	6.25E+02	3.17E+02	6.24E+02	C28H32O16	[M+H]+	Flavonoids	Flavonols	9.00E+00	9.00E+00	1.88E+05	9.00E+00	--	--	cluster1
Isorhamnetin-O-Hexoside-O-Hexoside	6.41E+02	4.79E+02	6.40E+02	C28H32O17	[M+H]+	Flavonoids	Flavonols	9.00E+00	9.00E+00	1.38E+06	1.30E+05	--	--	cluster1
Isorhamnetin-O-Hexoside-O-rhamnoside-O-rhamnoside	7.71E+02	4.79E+02	7.70E+02	C34H42O20	[M+H]+	Flavonoids	Flavonols	9.00E+00	9.00E+00	4.86E+04	9.00E+00	--	--	cluster1
Isorhamnetin-O-Hexoside-O-Hexoside-O-Rhamnoside	7.87E+02	3.17E+02	7.86E+02	C34H42O21	[M+H]+	Flavonoids	Flavonols	9.00E+00	9.00E+00	9.38E+05	1.97E+05	--	--	cluster1
Isoscutellarein	2.87E+02	1.53E+02	2.86E+02	C15H10O6	[M+H]+	Flavonoids	Flavonoid	9.00E+00	9.00E+00	3.15E+04	2.37E+04	--	--	cluster3
Aureusidin	2.87E+02	1.53E+02	2.86E+02	C15H10O6	[M+H]+	Flavonoids	Sinensetin	9.00E+00	9.00E+00	3.40E+04	2.04E+04	C08576	--	cluster3
Luteolin	2.85E+02	1.51E+02	2.86E+02	C15H10O6	[M-H]-	Flavonoids	Flavonoid	9.00E+00	9.00E+00	1.27E+05	1.02E+05	C01514	ko00941,ko00944,ko01100,ko01110	cluster3
2'-Hydroxyisoflavone	2.85E+02	1.99E+02	2.86E+02	C15H10O6	[M-H]-	Flavonoids	Isoflavones	9.00E+00	9.00E+00	1.00E+05	6.97E+04	--	--	cluster3
6,7,8-Tetrahydroxy-5-methoxyflavone	3.01E+02	2.86E+02	3.00E+02	C16H12O6	[M+H]+	Flavonoids	Flavonoid	9.00E+00	3.24E+03	1.12E+05	1.73E+05	--	--	cluster3
Quercetin	3.03E+02	1.37E+02	3.02E+02	C15H10O7	[M+H]+	Flavonoids	Flavonols	9.00E+00	9.00E+00	5.92E+04	2.41E+04	C00389	ko00941,ko00944,ko01100,ko01110	cluster3
Dihydroquercetin(Taxifolin)	3.03E+02	1.25E+02	3.04E+02	C15H12O7	[M-H]-	Flavonoids	Dihydroflavonol	2.44E+04	1.05E+04	1.10E+05	4.30E+04	C01617	ko00941,ko01100,ko01110	cluster3
Apigenin 5-O-glucoside	4.33E+02	2.71E+02	4.32E+02	C21H20O10	[M+H]+	Flavonoids	Flavonoid	2.24E+04	5.59E+04	1.74E+06	1.37E+06	--	--	cluster3
Genistein 7-Glucoside(Genistin)	4.31E+02	2.69E+02	4.32E+02	C21H20O10	[M-H]-	Flavonoids	Isoflavones	3.99E+03	1.34E+04	2.60E+05	4.01E+05	C09126	ko00943	cluster3
Apigenin-6-C-glucoside (Isovitexin)	4.31E+02	3.11E+02	4.32E+02	C21H20O10	[M-H]-	Flavonoids	Flavonoid carbonoside	7.88E+05	2.34E+06	2.28E+07	1.89E+07	C01714	ko00944	cluster3
Genistein 8-C-glucoside	4.33E+02	2.83E+02	4.32E+02	C21H20O10	[M+H]+	Flavonoids	Flavonoid carbonoside	8.07E+05	2.32E+06	3.57E+07	3.02E+07	C10420	--	cluster3
Quercetin 3-O-β-D-xylopyranoside	4.35E+02	3.03E+02	4.34E+02	C20H18O11	[M+H]+	Flavonoids	Flavonols	6.20E+04	6.41E+04	1.91E+06	1.36E+06	--	--	cluster3
Quercetin-3-O-α-L-arabinopyranoside(guaijaverin)	4.33E+02	3.00E+02	4.34E+02	C20H18O11	[M-H]-	Flavonoids	Flavonols	7.60E+04	7.17E+04	2.86E+06	2.16E+06	--	--	cluster3

Naringenin-7-O-glucoside	4.33E+02	2.71E+02	4.34E+02	C21H22O10	[M-H]-	Flavonoids	Dihydroflavone	9.00E+00	9.00E+00	5.14E+05	1.50E+05	--	--	cluster3
Luteolin-4'-O-β-D-glucoside	4.49E+02	2.87E+02	4.48E+02	C21H20O11	[M+H]+	Flavonoids	Flavonoid	6.07E+04	1.03E+05	9.34E+06	1.04E+07	--	--	cluster3
Quercetin-3-O-α-L-rhamnopyranoside	4.49E+02	2.87E+02	4.48E+02	C21H20O11	[M+H]+	Flavonoids	Flavonoid	1.88E+04	4.97E+04	3.35E+06	2.45E+06	--	--	cluster3
Kaempferol-3-O-galactoside (Trifolin)	4.47E+02	2.85E+02	4.48E+02	C21H20O11	[M-H]-	Flavonoids	Flavonols	5.10E+04	1.24E+05	7.34E+06	1.09E+07	C12626	ko00944	cluster3
Luteolin-6-C-glucoside (Isoorientin)	4.47E+02	3.57E+02	4.48E+02	C21H20O11	[M-H]-	Flavonoids	Flavonoid carbonoside	2.78E+05	1.39E+05	4.12E+07	3.49E+07	C01821	--	cluster3
Quercetin-3-O-α-L-rhamnoside(Quercitrin)	4.49E+02	3.03E+02	4.48E+02	C21H20O11	[M+H]+	Flavonoids	Flavonols	9.00E+00	9.00E+00	5.04E+05	1.75E+05	C01750	ko00944	cluster3
Luteolin-8-C-Glucoside (Orientin)	4.49E+02	2.99E+02	4.48E+02	C21H20O11	[M+H]+	Flavonoids	Flavonoid carbonoside	3.18E+05	2.12E+05	2.72E+07	2.36E+07	C10114	--	cluster3
Kaempferol-3-O-glucoside (Astragalin)	4.49E+02	2.87E+02	4.48E+02	C21H20O11	[M+H]+	Flavonoids	Flavonols	2.16E+04	5.16E+04	3.36E+06	2.54E+06	C12249	ko00944,ko01110	cluster3
Kaempferol-7-O-glucoside	4.47E+02	2.85E+02	4.48E+02	C21H20O11	[M-H]-	Flavonoids	Flavonols	7.56E+04	1.22E+05	1.99E+07	1.96E+07	--	--	cluster3
Luteolin 3'-O-β-D-glucoside	4.49E+02	2.87E+02	4.48E+02	C21H20O11	[M+H]+	Flavonoids	Flavonoid	5.84E+04	9.24E+04	9.38E+06	1.03E+07	--	--	cluster3
Cyanidin-3-O-glucoside (Kuromanin)	4.49E+02	2.87E+02	4.49E+02	C21H21O11+	[M]+	Flavonoids	Anthocyanins	1.04E+05	1.01E+05	3.75E+05	8.00E+05	C08604	ko00942	cluster3
Eriodictyol 7-O-glucoside	4.51E+02	2.89E+02	4.50E+02	C21H22O11	[M+H]+	Flavonoids	Dihydroflavone	1.33E+04	1.64E+04	2.44E+05	3.58E+05	--	--	cluster3
Pratensein 7-O-glucopyranoside	4.63E+02	3.01E+02	4.62E+02	C22H22O11	[M+H]+	Flavonoids	Flavonoid	9.00E+00	9.00E+00	3.49E+05	3.82E+05	--	--	cluster3
Chrysoeriol-7-O-glucoside	4.61E+02	2.99E+02	4.62E+02	C22H22O11	[M-H]-	Flavonoids	Flavonoid	2.20E+04	5.49E+04	2.34E+06	3.33E+06	--	--	cluster3
Diosmetin-6-C-glucoside	4.63E+02	4.27E+02	4.62E+02	C22H22O11	[M+H]+	Flavonoids	Flavonoid	9.00E+00	9.00E+00	1.76E+06	6.85E+05	--	--	cluster3
Isoscoparin	4.61E+02	3.41E+02	4.62E+02	C22H22O11	[M-H]-	Flavonoids	Flavonoid	3.79E+04	9.00E+00	8.43E+06	4.02E+06	C05990	--	cluster3
Peonidin-3-O-glucoside	4.63E+02	3.01E+02	4.63E+02	C22H23O11+	[M]+	Flavonoids	Anthocyanins	1.61E+04	9.00E+00	6.09E+05	4.51E+05	C12141	ko00942	cluster3
6-Hydroxyluteolin 5-glucoside	4.63E+02	3.01E+02	4.64E+02	C21H20O12	[M-H]-	Flavonoids	Flavonoid	7.44E+05	4.10E+05	4.58E+07	3.48E+07	--	--	cluster3
6-Hydroxykaempferol-7-O-glucoside	4.65E+02	3.03E+02	4.64E+02	C21H20O12	[M+H]+	Flavonoids	Flavonols	2.41E+04	1.59E+04	2.67E+05	1.62E+05	--	--	cluster3
Myricetin-O-rhamnoside	4.65E+02	3.03E+02	4.64E+02	C21H20O12	[M+H]+	Flavonoids	Flavonols	6.11E+04	3.24E+04	4.47E+06	2.67E+06	--	--	cluster3
Spiraeoside	4.63E+02	3.01E+02	4.64E+02	C21H20O12	[M-H]-	Flavonoids	Flavonols	7.80E+05	4.44E+05	5.33E+07	3.66E+07	--	--	cluster3
Quercetin-3-O-β-D-glucoside(Isoquercitrin)	4.63E+02	3.00E+02	4.64E+02	C21H20O12	[M-H]-	Flavonoids	Flavonols	5.32E+04	3.56E+04	6.21E+06	3.56E+06	C05623	ko00944,ko01100,ko01110	cluster3
isohyperoside	4.65E+02	3.03E+02	4.64E+02	C21H20O12	[M+H]+	Flavonoids	Flavonols	5.52E+04	3.75E+04	4.37E+06	2.55E+06	--	--	cluster3
Hesperetin 5-O-glucoside	4.63E+02	3.01E+02	4.64E+02	C22H24O11	[M-H]-	Flavonoids	Dihydroflavonol	7.34E+05	4.10E+05	5.26E+07	3.48E+07	--	--	cluster3
Quercetin-5-O-glucuronide	4.79E+02	3.03E+02	4.78E+02	C21H18O13	[M+H]+	Flavonoids	Flavonols	2.72E+04	9.00E+00	4.58E+06	3.92E+06	--	--	cluster3
Isorhamnetin-3-O-glucoside	4.79E+02	3.17E+02	4.78E+02	C22H22O12	[M+H]+	Flavonoids	Flavonols	2.87E+04	8.31E+03	9.14E+06	3.33E+06	--	--	cluster3
Isorhamnetin-7-O-glucoside	4.79E+02	3.17E+02	4.78E+02	C22H22O12	[M+H]+	Flavonoids	Flavonols	2.13E+04	1.06E+04	9.17E+06	3.34E+06	--	--	cluster3
Genistein 8-C-apiosyl(1→6)glucoside	5.65E+02	4.33E+02	5.64E+02	C26H28O14	[M+H]+	Flavonoids	Flavonoid carbonoside	2.50E+05	5.73E+05	6.54E+06	7.79E+06	--	--	cluster3
Kaempferol-O-Pentoside-O-hexoside	5.81E+02	2.87E+02	5.80E+02	C26H28O15	[M+H]+	Flavonoids	Flavonols	6.22E+04	1.10E+05	1.72E+06	1.87E+06	--	--	cluster3
6-C-Hexosyl luteolin O-pentoside	5.81E+02	4.31E+02	5.80E+02	C26H28O15	[M+H]+	Flavonoids	Flavonoid carbonoside	1.19E+04	8.91E+03	5.86E+05	6.73E+05	--	--	cluster3
Luteolin-7-O-β-D-glucosyl-6-C-α-L-arabinose	5.81E+02	3.83E+02	5.80E+02	C26H28O15	[M+H]+	Flavonoids	Flavonoid	9.00E+00	9.00E+00	8.90E+04	1.16E+05	--	--	cluster3
Naringenin-7-O-Rutinoside(Narirutin)	5.79E+02	2.71E+02	5.80E+02	C27H32O14	[M-H]-	Flavonoids	Dihydroflavone	1.00E+04	6.66E+03	9.88E+05	5.09E+05	C09793	--	cluster3
Luteolin-7-O-rutinoside	5.93E+02	4.73E+02	5.94E+02	C27H30O15	[M-H]-	Flavonoids	Flavonoid	1.95E+05	3.54E+05	7.38E+06	8.71E+06	--	--	cluster3
Luteolin 7-O-neohesperidoside(Lonicerin)	5.95E+02	4.49E+02	5.94E+02	C27H30O15	[M+H]+	Flavonoids	Flavonoid	4.06E+04	6.17E+04	7.14E+05	8.41E+05	C12630	ko00944	cluster3
Apigenin 6,8-C-diglucoside	5.95E+02	4.57E+02	5.94E+02	C27H30O15	[M+H]+	Flavonoids	Flavonoid	5.59E+05	1.08E+06	1.59E+07	1.82E+07	--	--	cluster3
Vitexin-2-O-D-glucopyranoside	5.95E+02	5.77E+02	5.94E+02	C27H30O15	[M+H]+	Flavonoids	Flavonoid carbonoside	1.20E+05	2.27E+05	2.94E+06	3.47E+06	--	--	cluster3
Kaempferol-3-O-glucoside-7-O-rhamnoside	5.95E+02	4.49E+02	5.94E+02	C27H30O15	[M+H]+	Flavonoids	Flavonols	3.85E+04	5.10E+04	7.30E+05	8.39E+05	--	--	cluster3
Kaempferol-3-O-neohesperidoside	5.95E+02	2.87E+02	5.94E+02	C27H30O15	[M+H]+	Flavonoids	Flavonoid	2.17E+03	1.15E+04	4.96E+05	4.96E+05	--	--	cluster3
Kaempferol-3-O-rutinoside(Nicotiflorin)	5.93E+02	2.85E+02	5.94E+02	C27H30O15	[M-H]-	Flavonoids	Flavonols	2.53E+05	3.27E+05	1.04E+07	1.27E+07	C21833	ko00944,ko01110	cluster3
Quercetin-3-arabinoylglucoside	5.95E+02	3.00E+02	5.96E+02	C26H28O16	[M-H]-	Flavonoids	Flavonols	1.06E+04	9.00E+00	1.53E+05	8.90E+04	--	--	cluster3
Eriocitrin	5.95E+02	2.87E+02	5.96E+02	C27H32O15	[M-H]-	Flavonoids	Dihydroflavone	8.75E+03	9.00E+00	2.01E+05	8.85E+04	C09732	--	cluster3
Neodiosmin (Diosmetin-7-O-Neohesperidoside)	6.09E+02	4.63E+02	6.08E+02	C28H32O15	[M+H]+	Flavonoids	Flavonoid	1.63E+04	2.34E+04	1.14E+06	1.59E+06	--	--	cluster3
Diosmin	6.09E+02	3.01E+02	6.08E+02	C28H32O15	[M+H]+	Flavonoids	Flavonoid	5.50E+03	1.05E+04	4.56E+05	5.81E+05	C10039	--	cluster3
Chrysoeriol-7-O-rutinoside	6.07E+02	2.99E+02	6.08E+02	C28H32O15	[M-H]-	Flavonoids	Flavonoid	5.07E+04	7.43E+04	5.03E+06	6.29E+06	--	--	cluster3
Luteolin-7-O-β-D-gentiobioside	6.11E+02	2.87E+02	6.10E+02	C27H30O16	[M+H]+	Flavonoids	Flavonoid	6.92E+04	1.70E+05	3.02E+06	3.46E+06	--	--	cluster3
Quercetin-O-feruloyl-Pentoside	6.11E+02	3.03E+02	6.10E+02	C30H26O14	[M+H]+	Flavonoids	Flavonols	8.46E+04	6.91E+04	1.16E+06	8.16E+05	--	--	cluster3
C-Hexosyl-luteolin O-hexoside	6.11E+02	4.31E+02	6.10E+02	C27H30O16	[M+H]+	Flavonoids	Flavonoid carbonoside	1.66E+04	1.06E+04	7.31E+05	6.00E+05	--	--	cluster3
Quercetin-3-O-neohesperidoside	6.11E+02	4.65E+02	6.10E+02	C27H30O16	[M+H]+	Flavonoids	Flavonols	1.77E+05	1.36E+05	1.70E+06	1.11E+06	--	--	cluster3
6-C-Hexosyl-luteolin O-hexoside	6.11E+02	4.31E+02	6.10E+02	C27H30O16	[M+H]+	Flavonoids	Flavonoid carbonoside	9.00E+00	9.19E+03	1.13E+05	1.38E+05	--	--	cluster3
Quercetin-3-O-robinobioside	6.09E+02	3.00E+02	6.10E+02	C27H30O16	[M-H]-	Flavonoids	Flavonols	4.43E+04	3.07E+04	1.51E+06	1.06E+06	--	--	cluster3
Luteolin-8-C-hexosyl-O-hexoside	6.11E+02	4.65E+02	6.10E+02	C27H30O16	[M+H]+	Flavonoids	Flavonoid	2.12E+05	1.42E+05	1.85E+06	1.24E+06	--	--	cluster3
Isoluteolin-6,8-di-C-glucoside	6.11E+02	4.91E+02	6.10E+02	C27H30O16	[M+H]+	Flavonoids	Flavonoid	1.19E+04	9.93E+03	3.63E+05	3.50E+05	--	--	cluster3
Quercetin-3-O-rutinoside (Rutin)	6.09E+02	3.01E+02	6.10E+02	C27H30O16	[M-H]-	Flavonoids	Flavonols	5.56E+04	3.72E+04	1.66E+06	1.18E+06	C05625	ko00944,ko01100,ko01110	cluster3
Quercetin glu-rha	6.11E+02	3.03E+02	6.10E+02	C27H30O16	[M+H]+	Flavonoids	Flavonols	8.60E+04	7.59E+04	1.13E+06	7.53E+05	--	--	cluster3
Hesperidin	6.09E+02	3.01E+02	6.10E+02	C28H34O15	[M-H]-	Flavonoids	Dihydroflavone	1.46E+04	9.00E+00	1.66E+05	1.38E+05	C09755	--	cluster3
Hesperetin 7-O-neohesperidoside(Neohesperidin)	6.09E+02	3.01E+02	6.10E+02	C28H34O15	[M-H]-	Flavonoids	Dihydroflavone	2.57E+04	1.17E+04	6.42E+05	6.89E+05	C09806	ko00941	cluster3
Eriodictiol C-hexosyl-O-hexoside	6.13E+02	4.51E+02	6.12E+02	C27H32O16	[M+H]+	Flavonoids	Dihydroflavone	1.37E+04	1.52E+04	1.28E+05	1.59E+05	--	--	cluster3
Quercetin 3-O-(6″-galloyl)-β-D-glucopyranoside	6.17E+02	3.03E+02	6.16E+02	C28H24O16	[M+H]+	Flavonoids	Flavonoid	9.00E+00	9.00E+00	1.13E+05	7.80E+04	--	--	cluster3
Isochrysoeriol O-dihexoside	6.25E+02	4.63E+02	6.24E+02	C28H32O16	[M+H]+	Flavonoids	Flavonoid	9.00E+00	9.00E+00	1.71E+05	5.33E+04	--	--	cluster3
Chrysoeriol-O-hexosyl-O-hexoside	6.25E+02	3.02E+02	6.24E+02	C28H32O16	[M+H]+	Flavonoids	Flavonoid	8.03E+03	9.00E+00	3.87E+05	1.41E+05	--	--	cluster3

6-C-Hexosyl chrysoeriol O-hexoside	6.25E+02	6.07E+02	6.24E+02	C28H32O16	[M+H]+	Flavonoids	Flavonoid carbonoside	9.00E+00	9.00E+00	8.91E+04	3.00E+04	--	--	cluster3
2'-Hydoxy,5-methoxy Genistein-O-rhamnosyl-glucoside	6.25E+02	3.17E+02	6.24E+02	C28H32O16	[M+H]+	Flavonoids	Isoflavones	7.19E+03	2.84E+03	2.69E+06	9.38E+05	--	--	cluster3
Chrysoeriol-6,8-di-C-glucoside	6.25E+02	6.07E+02	6.24E+02	C28H32O16	[M+H]+	Flavonoids	Flavonoid carbonoside	9.00E+00	9.00E+00	1.11E+05	7.80E+04	--	--	cluster3
Isorhamnetin-O-rutinoside	6.25E+02	4.63E+02	6.24E+02	C28H32O16	[M+H]+	Flavonoids	Flavonols	1.25E+04	9.51E+03	2.00E+05	1.24E+05	--	--	cluster3
6-Hydroxykaempferol-3,6-O-Diglucoside	6.27E+02	3.03E+02	6.26E+02	C27H30O17	[M+H]+	Flavonoids	Flavonols	8.39E+04	4.62E+04	5.84E+05	2.24E+05	--	--	cluster3
Chrysoeriol-C-pentosyl-O-hexosyl-O-hexoside	7.55E+02	4.47E+02	7.56E+02	C33H40O20	[M-H]-	Flavonoids	Flavonoid	1.68E+04	1.93E+04	1.15E+07	1.43E+07	--	--	cluster3
Quercetin-O-rhamnoside-O-Hexoside-O-rhamnoside	7.57E+02	4.49E+02	7.56E+02	C33H40O20	[M+H]+	Flavonoids	Flavonols	1.19E+05	1.44E+05	3.78E+06	4.42E+06	--	--	cluster3
Kaempferol-3-O-neohesperidoside-7-glucoside	7.57E+02	7.39E+02	7.56E+02	C33H40O20	[M+H]+	Flavonoids	Flavonols	4.40E+03	9.00E+00	3.10E+05	4.26E+05	--	--	cluster3
Quercetin-O-rutinoside-hexose	7.71E+02	6.09E+02	7.72E+02	C33H40O21	[M-H]-	Flavonoids	Flavonols	9.00E+00	9.00E+00	1.52E+06	9.48E+05	--	--	cluster3
6-Hydroxykaempferol-3,7,6-O-triglycoside	7.89E+02	3.03E+02	7.88E+02	C33H40O22	[M+H]+	Flavonoids	Flavonols	1.02E+04	4.11E+03	2.23E+05	6.36E+04	--	--	cluster3
Hesperetin C-hexosyl-O-hexosyl-O-hexoside	7.89E+02	6.27E+02	7.88E+02	C34H44O21	[M+H]+	Flavonoids	Dihydroflavonol	8.68E+03	2.83E+03	1.38E+05	4.32E+04	--	--	cluster3
Apigenin-7-O-(6'-O-acetyl)-β-D-glucoside	4.75E+02	2.71E+02	4.74E+02	C23H22O11	[M+H]+	Flavonoids	Flavonoid	7.65E+03	8.15E+03	5.03E+03	8.24E+03	--	--	cluster5
6"-O-Acetylgenistin	4.75E+02	2.71E+02	4.74E+02	C23H22O11	[M+H]+	Flavonoids	Isoflavones	7.23E+03	7.39E+03	4.51E+03	7.02E+03	--	--	cluster5
Epicatechin	2.91E+02	1.39E+02	2.90E+02	C15H14O6	[M+H]+	Flavonoids	Flavanols	6.51E+05	7.53E+05	1.20E+04	1.81E+04	C09727	ko00941,ko01100,ko01110	cluster6
Catechin	2.89E+02	2.45E+02	2.90E+02	C15H14O6	[M-H]-	Flavonoids	Flavanols	2.34E+05	3.33E+05	1.25E+04	2.12E+04	C06562	ko00941,ko01110	cluster6
Gallocatechin 3-O-gallate	4.57E+02	1.69E+02	4.58E+02	C22H18O11	[M-H]-	Flavonoids	Flavanols	7.09E+04	1.23E+05	2.16E+04	9.00E+00	--	--	cluster6
Luteolin-7-O-glucuronide	4.63E+02	2.87E+02	4.62E+02	C21H18O12	[M+H]+	Flavonoids	Flavonoid	2.10E+04	2.51E+04	8.04E+03	6.90E+03	--	--	cluster6
Luteolin-O-eudesmic acid-O-hexoside	6.41E+02	2.85E+02	6.42E+02	C31H30O15	[M-H]-	Flavonoids	Flavonoid	5.44E+04	3.56E+04	9.00E+00	9.00E+00	--	--	cluster6
Catechin gallate	4.41E+02	1.69E+02	4.42E+02	C22H18O10	[M-H]-	Flavonoids	Flavanols	2.43E+04	1.69E+04	2.46E+04	9.00E+00	--	--	cluster8
Tangeretin	3.73E+02	3.43E+02	3.72E+02	C20H20O7	[M+H]+	Flavonoids	Flavonols	4.86E+04	1.62E+04	2.30E+04	1.04E+04	C10190	--	cluster9
Nobiletin	4.03E+02	3.73E+02	4.02E+02	C21H22O8	[M+H]+	Flavonoids	Flavonoid	2.41E+04	8.92E+03	1.04E+04	5.78E+03	C10112	--	cluster9
Limocitrin-3-O-(3-hydroxy-3-methylglutarate)-glucoside	6.53E+02	3.47E+02	6.52E+02	C29H32O17	[M+H]+	Flavonoids	Flavonols	8.44E+05	3.80E+05	1.80E+05	1.80E+05	--	--	cluster9
Esculin(6,7-DihydroxyCoumarin-6-glucoside)	3.39E+02	1.77E+02	3.40E+02	C15H16O9	[M-H]-	Lignans and Coumarins	Coumarins	1.05E+04	9.00E+00	7.01E+04	9.00E+00	C09264	--	cluster1
Pinoresinol	3.57E+02	1.51E+02	3.58E+02	C20H22O6	[M-H]-	Lignans and Coumarins	Lignans	4.13E+03	9.00E+00	2.08E+05	1.87E+04	C05366	ko00998,ko01110	cluster1
Medioresinol	3.87E+02	1.81E+02	3.88E+02	C21H24O7	[M-H]-	Lignans and Coumarins	Lignans	9.00E+00	9.00E+00	5.77E+04	4.73E+03	--	--	cluster1
6-Hydroxy-4-methylcoumarin	1.75E+02	1.31E+02	1.76E+02	C10H8O3	[M-H]-	Lignans and Coumarins	Coumarins	1.72E+05	9.00E+00	3.11E+04	9.00E+00	--	--	cluster9
Undecylic Acid	1.85E+02	1.85E+02	1.86E+02	C11H22O2	[M-H]-	Lipids	Free fatty acids	2.77E+05	2.64E+05	2.89E+05	2.46E+05	--	--	cluster1
10,16-Dihydroxy-palmitic acid	2.87E+02	2.41E+02	2.88E+02	C16H32O4	[M-H]-	Lipids	Free fatty acids	1.35E+04	5.88E+03	4.21E+04	4.14E+04	C08285	ko00073	cluster3
LysoPC 16:1	4.94E+02	1.84E+02	4.93E+02	C24H48NO7P	[M+H]+	Lipids	LPC	4.89E+05	6.10E+05	4.17E+05	1.01E+06	--	--	cluster4
LysoPC 18:3	5.18E+02	1.84E+02	5.17E+02	C26H48NO7P	[M+H]+	Lipids	LPC	4.90E+06	5.98E+06	5.79E+06	8.90E+06	--	--	cluster4
LysoPE 16:0(2n isomer)	4.54E+02	3.13E+02	4.53E+02	C21H44NO7P	[M+H]+	Lipids	LPE	6.59E+06	1.09E+07	2.06E+06	5.27E+06	--	--	cluster5
LysoPC 15:1	4.80E+02	1.84E+02	4.79E+02	C23H46NO7P	[M+H]+	Lipids	LPC	1.78E+05	1.89E+05	1.03E+05	1.77E+05	--	--	cluster5
LysoPE 18:1	4.80E+02	3.39E+02	4.79E+02	C23H46NO7P	[M+H]+	Lipids	LPE	2.48E+06	4.93E+06	1.43E+05	9.75E+05	--	--	cluster5
LysoPC 15:0	4.82E+02	1.84E+02	4.81E+02	C23H48NO7P	[M+H]+	Lipids	LPC	6.30E+04	1.05E+05	3.88E+04	5.50E+04	--	--	cluster5
LysoPC 16:0	4.96E+02	1.84E+02	4.95E+02	C24H50NO7P	[M+H]+	Lipids	LPC	8.36E+06	1.19E+07	5.10E+06	1.19E+07	--	--	cluster5
LysoPC 17:0	5.10E+02	1.84E+02	5.09E+02	C25H52NO7P	[M+H]+	Lipids	LPC	4.97E+04	8.60E+04	3.34E+04	8.08E+04	--	--	cluster5
LysoPC 18:2	5.20E+02	1.84E+02	5.19E+02	C26H50NO7P	[M+H]+	Lipids	LPC	7.60E+06	8.84E+06	4.39E+06	9.63E+06	--	--	cluster5
LysoPC 18:1	5.22E+02	1.84E+02	5.21E+02	C26H52NO7P	[M+H]+	Lipids	LPC	1.42E+07	2.81E+07	4.04E+06	1.72E+07	--	--	cluster5
LysoPC 18:0	5.24E+02	1.84E+02	5.23E+02	C26H54NO7P	[M+H]+	Lipids	LPC	5.24E+05	9.75E+05	3.22E+05	1.07E+06	--	--	cluster5
Hexadecylsphingosine	2.74E+02	2.56E+02	2.73E+02	C16H35NO2	[M+H]+	Lipids	Sphingolipids	8.97E+05	7.99E+05	3.74E+05	4.38E+05	--	--	cluster6
Elaidic Acid	2.81E+02	2.81E+02	2.82E+02	C18H34O2	[M-H]-	Lipids	Free fatty acids	3.36E+07	2.79E+07	1.67E+07	1.94E+07	--	--	cluster6
11-Octadecanoic acid(Vaccenic acid)	2.81E+02	2.81E+02	2.82E+02	C18H34O2	[M-H]-	Lipids	Free fatty acids	1.24E+07	9.71E+06	5.68E+06	6.49E+06	--	--	cluster6
Stearic Acid	2.83E+02	2.83E+02	2.84E+02	C18H36O2	[M-H]-	Lipids	Free fatty acids	4.98E+07	4.74E+07	4.11E+07	4.57E+07	--	--	cluster6
LysoPE 14:0	4.26E+02	2.85E+02	4.25E+02	C19H40NO7P	[M+H]+	Lipids	LPE	4.07E+05	3.90E+05	8.90E+04	1.98E+05	--	--	cluster6
LysoPE 18:2	4.78E+02	3.37E+02	4.77E+02	C23H44NO7P	[M+H]+	Lipids	LPE	1.27E+07	1.47E+07	1.43E+06	6.16E+06	--	--	cluster6
LysoPE 18:0(2n isomer)	4.82E+02	3.41E+02	4.81E+02	C23H48NO7P	[M+H]+	Lipids	LPE	1.18E+04	1.27E+04	9.00E+00	4.53E+03	--	--	cluster6
Lauric acid	1.99E+02	1.81E+02	2.00E+02	C12H24O2	[M-H]-	Lipids	Free fatty acids	3.03E+03	1.99E+03	2.47E+03	1.82E+03	C02679	ko00061,ko01100	cluster8
Myristic Acid	2.27E+02	2.27E+02	2.28E+02	C14H28O2	[M-H]-	Lipids	Free fatty acids	1.54E+07	9.79E+06	1.03E+07	1.03E+07	C06424	ko00061,ko01100	cluster9
Pentadecanoic Acid	2.41E+02	2.41E+02	2.42E+02	C15H30O2	[M-H]-	Lipids	Free fatty acids	2.60E+05	2.40E+05	2.23E+05	2.42E+05	--	--	cluster9
Cis-10-Heptadecenoic Acid	2.67E+02	2.67E+02	2.68E+02	C17H32O2	[M-H]-	Lipids	Free fatty acids	3.83E+05	1.43E+05	2.15E+05	2.01E+05	--	--	cluster9
Punicic acid	2.79E+02	9.51E+01	2.78E+02	C18H30O2	[M+H]+	Lipids	Free fatty acids	1.11E+06	4.99E+05	2.46E+05	2.67E+05	C08364	--	cluster9
9,10-EODE	2.95E+02	2.77E+02	2.96E+02	C18H32O3	[M-H]-	Lipids	Free fatty acids	1.71E+06	8.61E+05	4.23E+05	4.73E+05	--	--	cluster9
13-Hydroxy-9,11-octadecadienoic acid	2.95E+02	1.95E+02	2.96E+02	C18H32O3	[M-H]-	Lipids	Free fatty acids	8.87E+05	3.74E+05	2.05E+05	2.22E+05	--	--	cluster9
9-Hydroxy-12-oxo-10-octadecenoic acid	3.11E+02	2.23E+02	3.12E+02	C18H32O4	[M-H]-	Lipids	Free fatty acids	2.32E+05	1.73E+04	9.07E+04	2.85E+04	--	--	cluster9
9,10,13-Trihyroxy-11-octadecadienoic acid	3.29E+02	2.29E+02	3.30E+02	C18H34O5	[M-H]-	Lipids	Free fatty acids	9.43E+05	3.07E+05	4.77E+05	2.94E+05	--	--	cluster9
Monopalmitin	3.31E+02	3.13E+02	3.30E+02	C19H38O4	[M+H]+	Lipids	Glycerol ester	9.18E+04	5.63E+04	4.60E+04	5.08E+04	--	--	cluster9
Glyceryl linoleate	3.55E+02	3.37E+02	3.54E+02	C21H38O4	[M+H]+	Lipids	Glycerol ester	3.67E+04	2.07E+04	1.26E+04	1.36E+04	--	--	cluster9
MAG(18:2)	3.55E+02	2.63E+02	3.54E+02	C21H38O4	[M+H]+	Lipids	Glycerol ester	5.73E+05	1.96E+05	2.32E+05	2.32E+05	--	--	cluster9
MAG(18:1)isomer1	3.57E+02	2.65E+02	3.56E+02	C21H40O4	[M+H]+	Lipids	Glycerol ester	1.42E+05	7.52E+04	3.54E+04	3.51E+04	--	--	cluster9
LysoPC 14:0(2n isomer)	4.68E+02	1.84E+02	4.67E+02	C22H46NO7P	[M+H]+	Lipids	LPC	2.71E+05	2.13E+05	1.66E+05	2.27E+05	--	--	cluster9

γ-Linolenic Acid	2.77E+02	2.77E+02	2.78E+02	C18H30O2	[M-H]-	Lipids	Free fatty acids	5.79E+06	2.39E+06	5.65E+06	3.30E+06	C06426	ko00591,ko01040,ko01100	cluster10
12,13-EODE	2.95E+02	1.95E+02	2.96E+02	C18H32O3	[M-H]-	Lipids	Free fatty acids	1.60E+05	5.32E+04	1.92E+05	1.19E+05	--	--	cluster10
LysoPC 16:2(2n isomer)	4.92E+02	1.84E+02	4.91E+02	C24H46NO7P	[M+H]+	Lipids	LPC	4.21E+04	1.28E+04	3.46E+04	3.49E+04	--	--	cluster10
1-Methyladenine	1.50E+02	1.33E+02	1.49E+02	C6H7N5	[M+H]+	Nucleotides and derivatives	Nucleotides and derivatives	6.76E+04	3.25E+04	1.54E+05	5.49E+04	C02216	--	cluster1
2-Hydroxy-6-aminopurine	1.52E+02	1.35E+02	1.51E+02	C5H5N5O	[M+H]+	Nucleotides and derivatives	Nucleotides and derivatives	2.00E+05	1.37E+05	4.37E+05	1.44E+05	--	--	cluster1
Guanine	1.52E+02	1.35E+02	1.51E+02	C5H5N5O	[M+H]+	Nucleotides and derivatives	Nucleotides and derivatives	1.26E+05	1.59E+05	2.39E+05	5.64E+04	C00242	ko00230,ko01100	cluster1
Uridine 5'-monophosphate	3.23E+02	2.11E+02	3.24E+02	C9H13N2O9P	[M-H]-	Nucleotides and derivatives	Nucleotides and derivatives	1.78E+05	9.33E+04	2.53E+05	1.04E+05	C00105	ko00240,ko01100	cluster1
3'-Aenylic acid	3.46E+02	2.11E+02	3.47E+02	C10H14N5O7P	[M-H]-	Nucleotides and derivatives	Nucleotides and derivatives	3.47E+04	1.93E+04	5.25E+04	1.78E+04	C01367	ko00230,ko01100	cluster1
Adenosine 5'-monophosphate	3.48E+02	1.36E+02	3.47E+02	C10H14N5O7P	[M+H]+	Nucleotides and derivatives	Nucleotides and derivatives	4.16E+06	2.40E+06	8.30E+06	3.10E+06	C00020	ko00230,ko00908,ko01100,ko01110	cluster1
Guanosine 5'-monophosphate	3.64E+02	1.52E+02	3.63E+02	C10H14N5O8P	[M+H]+	Nucleotides and derivatives	Nucleotides and derivatives	1.07E+05	6.69E+04	1.85E+05	6.12E+04	C00144	ko00230,ko01100	cluster1
Nicotinic acid adenine dinucleotide	6.64E+02	1.36E+02	6.63E+02	C21H27N7O14P2	[M+H]+	Nucleotides and derivatives	Nucleotides and derivatives	6.05E+05	6.38E+05	8.24E+05	5.70E+05	C00003	ko00190,ko00730,ko00760,ko01100	cluster1
Cytosine	1.12E+02	9.50E+01	1.11E+02	C4H5N3O	[M+H]+	Nucleotides and derivatives	Nucleotides and derivatives	1.53E+05	1.88E+05	5.30E+05	1.04E+06	C00380	ko00240,ko01100	cluster3
Thymine	1.27E+02	1.10E+02	1.26E+02	C5H6N2O2	[M+H]+	Nucleotides and derivatives	Nucleotides and derivatives	9.00E+00	9.00E+00	1.17E+05	1.00E+05	C00178	ko00240,ko01100	cluster3
Adenine	1.36E+02	1.19E+02	1.35E+02	C5H5N5	[M+H]+	Nucleotides and derivatives	Nucleotides and derivatives	5.14E+06	6.08E+06	8.05E+06	1.43E+07	C00147	ko00230,ko00908,ko01100	cluster3
Hypoxanthine	1.37E+02	1.19E+02	1.36E+02	C5H4N4O	[M+H]+	Nucleotides and derivatives	Nucleotides and derivatives	2.86E+04	4.03E+04	1.98E+05	2.57E+05	C00262	ko00230,ko01100	cluster3
Thymidine	2.43E+02	1.27E+02	2.42E+02	C10H14N2O5	[M+H]+	Nucleotides and derivatives	Nucleotides and derivatives	8.01E+03	4.17E+03	7.63E+04	5.57E+04	C00214	ko00240,ko01100	cluster3
Cytidine	2.44E+02	1.12E+02	2.43E+02	C9H13N3O5	[M+H]+	Nucleotides and derivatives	Nucleotides and derivatives	6.82E+05	7.40E+05	1.82E+06	3.80E+06	C00475	ko00240,ko01100,ko02010	cluster3
β-Pseudouridine	2.43E+02	1.53E+02	2.44E+02	C9H12N2O6	[M-H]-	Nucleotides and derivatives	Nucleotides and derivatives	2.47E+05	2.04E+05	2.66E+05	3.13E+05	C02067	ko00240,ko01100	cluster3
Uridine	2.43E+02	1.10E+02	2.44E+02	C9H12N2O6	[M-H]-	Nucleotides and derivatives	Nucleotides and derivatives	2.74E+06	2.14E+06	3.25E+06	4.16E+06	C00299	ko00240,ko01100,ko02010	cluster3
Deoxyadenosine	2.52E+02	1.36E+02	2.51E+02	C10H13N5O3	[M+H]+	Nucleotides and derivatives	Nucleotides and derivatives	2.13E+05	4.73E+04	2.36E+06	1.26E+06	C00559	ko00230,ko01100,ko02010	cluster3
5-Methyluridine	2.57E+02	1.24E+02	2.58E+02	C10H14N2O6	[M-H]-	Nucleotides and derivatives	Nucleotides and derivatives	2.00E+04	1.23E+04	4.26E+05	3.07E+05	--	--	cluster3
Adenosine	2.68E+02	1.36E+02	2.67E+02	C10H13N5O4	[M+H]+	Nucleotides and derivatives	Nucleotides and derivatives	1.37E+06	8.20E+05	4.75E+06	4.95E+06	C00212	ko00230,ko01100,ko02010	cluster3
Deoxyguanosine	2.68E+02	1.52E+02	2.67E+02	C10H13N5O4	[M+H]+	Nucleotides and derivatives	Nucleotides and derivatives	2.67E+04	8.50E+03	1.87E+05	7.56E+04	C00330	ko00230,ko01100,ko02010	cluster3
Guanosine	2.84E+02	1.52E+02	2.83E+02	C10H13N5O5	[M+H]+	Nucleotides and derivatives	Nucleotides and derivatives	4.03E+06	3.52E+06	1.04E+07	7.57E+06	C00387	ko00230,ko01100,ko02010	cluster3
Xanthosine	2.83E+02	1.51E+02	2.84E+02	C10H12N4O6	[M-H]-	Nucleotides and derivatives	Nucleotides and derivatives	4.79E+06	4.65E+06	1.37E+07	8.39E+06	C01762	ko00230,ko00232,ko01100,ko01110,ko02010	cluster3
β-Nicotinamide mononucleotide	3.35E+02	1.23E+02	3.34E+02	C11H15N2O8P	[M+H]+	Nucleotides and derivatives	Nucleotides and derivatives	1.65E+05	2.34E+05	2.47E+05	4.79E+05	C00455	ko00760,ko01100	cluster4
Xanthine	1.51E+02	1.51E+02	1.52E+02	C5H4N4O2	[M-H]-	Nucleotides and derivatives	Nucleotides and derivatives	9.00E+00	4.29E+04	1.18E+04	3.20E+04	C00385	ko00230,ko00232,ko01100,ko01110	cluster5
2'-Deoxyadenosine-5'-monophosphate	3.32E+02	1.36E+02	3.31E+02	C10H14N5O6P	[M+H]+	Nucleotides and derivatives	Nucleotides and derivatives	1.32E+05	1.58E+05	1.16E+05	8.10E+04	C00360	ko00230,ko01100	cluster7
Allopurinol	1.37E+02	1.10E+02	1.36E+02	C5H4N4O	[M+H]+	Nucleotides and derivatives	Nucleotides and derivatives	4.77E+04	2.80E+04	4.43E+04	2.65E+04	--	--	cluster8
Uridine 5'-diphospho-D-glucose	5.65E+02	3.23E+02	5.66E+02	C15H24N2O17P2	[M-H]-	Nucleotides and derivatives	Nucleotides and derivatives	4.47E+04	1.66E+04	3.16E+04	6.14E+03	C00029	ko00040,ko00052,ko00053,ko00240,ko00500,ko00520,ko00561,ko00908,ko01100	cluster8
9-(β-D-Arabinofuranosyl)hypoxanthine	2.67E+02	1.35E+02	2.68E+02	C10H12N4O5	[M-H]-	Nucleotides and derivatives	Nucleotides and derivatives	8.31E+05	4.52E+05	5.20E+05	3.01E+05	--	--	cluster9
N6-Succinyl Adenosine	3.84E+02	2.52E+02	3.83E+02	C14H17N5O8	[M+H]+	Nucleotides and derivatives	Nucleotides and derivatives	8.49E+06	2.91E+06	2.46E+06	2.62E+06	--	--	cluster9
2-(Dimethylamino)guanosine	3.12E+02	1.80E+02	3.11E+02	C12H17N5O5	[M+H]+	Nucleotides and derivatives	Nucleotides and derivatives	8.17E+04	1.69E+04	1.19E+05	4.28E+04	--	--	cluster10
Cytidine 5'-monophosphate(Cytidylic acid)	3.24E+02	1.12E+02	3.23E+02	C9H14N3O8P	[M+H]+	Nucleotides and derivatives	Nucleotides and derivatives	2.03E+05	1.38E+05	2.77E+05	1.82E+05	C00055	ko00240,ko01100	cluster10
6-Aminocaproic acid	1.32E+02	6.90E+01	1.31E+02	C6H13NO2	[M+H]+	Organic acids	Organic acids	3.45E+06	9.74E+05	1.38E+07	4.66E+06	C02378	ko01100	cluster1
Trans,trans-Muconic acid	1.41E+02	5.90E+01	1.42E+02	C6H6O4	[M-H]-	Organic acids	Organic acids	1.04E+06	1.19E+06	1.41E+06	7.90E+05	--	--	cluster1
Trans-Citridic acid	1.75E+02	9.90E+01	1.74E+02	C6H6O6	[M+H]+	Organic acids	Organic acids	1.71E+05	2.00E+05	2.02E+05	1.80E+05	C02341	ko00660,ko01100	cluster2
Fumaric acid	1.15E+02	7.10E+01	1.16E+02	C4H4O4	[M-H]-	Organic acids	Organic acids	9.00E+00	9.00E+00	2.87E+05	2.74E+05	C00122	ko00020,ko00190,ko00220,ko00250,ko00350,ko00360,ko00620,ko00650,ko00760,ko01100,ko01110,ko01200	cluster3
4-Guanidinobutyric acid	1.46E+02	8.70E+01	1.45E+02	C5H11N3O2	[M+H]+	Organic acids	Organic acids	2.76E+05	1.71E+05	6.75E+05	7.52E+05	C01035	ko00330,ko01100	cluster3
Adipic Acid	1.45E+02	1.01E+02	1.46E+02	C6H10O4	[M-H]-	Organic acids	Organic acids	1.14E+06	5.41E+05	2.03E+06	1.89E+06	C06104	ko01100	cluster3
(S)-(-)-2-Hydroxyisocaproic acid	1.31E+02	8.50E+01	1.32E+02	C6H12O3	[M-H]-	Organic acids	Organic acids	1.81E+06	2.65E+06	9.99E+05	1.82E+06	--	--	cluster5
D-Xylonic acid	1.65E+02	7.50E+01	1.66E+02	C5H10O6	[M-H]-	Organic acids	Organic acids	4.03E+06	4.58E+06	1.98E+06	4.18E+06	C00502	ko00040,ko01100	cluster5
D-Galacturonic acid(Gal A)	1.93E+02	7.28E+01	1.94E+02	C6H10O7	[M-H]-	Organic acids	Organic acids	1.17E+05	1.26E+05	4.52E+04	4.01E+04	C00333	ko00040,ko00053,ko00520,ko01100,ko02010	cluster6
2-Aminoethanesulfonic acid	1.24E+02	8.00E+01	1.25E+02	C2H7NO3S	[M-H]-	Organic acids	Organic acids	6.13E+04	8.43E+04	6.92E+04	4.90E+04	C00245	ko00430,ko00920,ko01100,ko02010	cluster7
3-Hydroxy-3-methyl butyric acid	1.17E+02	5.90E+01	1.18E+02	C5H10O3	[M-H]-	Organic acids	Organic acids	4.51E+05	2.91E+05	2.05E+05	1.02E+05	--	--	cluster8
Anchoic Acid	1.87E+02	1.25E+02	1.88E+02	C9H16O4	[M-H]-	Organic acids	Organic acids	4.05E+05	3.37E+05	2.79E+05	2.08E+05	C08261	--	cluster8
Methylmalonic acid	1.17E+02	7.30E+01	1.18E+02	C4H6O4	[M-H]-	Organic acids	Organic acids	1.58E+07	6.92E+06	3.46E+06	3.91E+06	C02170	ko00240,ko00280,ko00640,ko01100	cluster9

Succinic acid	1.17E+02	7.30E+01	1.18E+02	C4H6O4	[M-H]-	Organic acids	Organic acids	1.57E+07	6.88E+06	3.51E+06	3.87E+06	C00042	ko00020,ko00190,ko00250,ko00310,ko00350,ko00360,ko00620,ko00630,ko00640,ko00650,ko00760,ko00920,ko01100,ko01110,ko01200	cluster9
L-(-)-Malic acid	1.33E+02	7.10E+01	1.34E+02	C4H6O5	[M-H]-	Organic acids	Organic acids	4.43E+07	2.04E+07	1.38E+07	7.26E+06	C00497	ko00650,ko01100	cluster9
4-Acetamidobutyric acid	1.46E+02	8.60E+01	1.45E+02	C6H11NO3	[M+H]+	Organic acids	Organic acids	9.10E+05	3.11E+05	1.03E+05	9.74E+04	C02946	ko00330,ko01100	cluster9
Citramalate	1.47E+02	8.71E+01	1.48E+02	C5H8O5	[M-H]-	Organic acids	Organic acids	6.52E+04	2.94E+04	2.57E+04	4.41E+04	C00815	--	cluster9
Citric Acid	1.91E+02	1.11E+02	1.92E+02	C6H8O7	[M-H]-	Organic acids	Organic acids	1.39E+07	5.27E+06	7.50E+06	1.09E+07	C00158	ko00020,ko00250,ko00630,ko01100,ko01110,ko01200,ko01210,ko01230	cluster9
Quinic Acid	1.91E+02	8.50E+01	1.92E+02	C7H12O6	[M-H]-	Organic acids	Organic acids	1.73E+07	9.97E+06	8.48E+06	1.39E+07	C00296	ko00400,ko01100	cluster9
O-Phosphorylethanolamine	1.40E+02	7.90E+01	1.41E+02	C2H8NO4P	[M-H]-	Others	Others	4.34E+04	2.66E+04	6.92E+04	3.38E+04	C00346	ko00564,ko00600,ko01100	cluster1
Turanose	3.65E+02	2.03E+02	3.64E+02	C12H21O11Na	[M+H]+	Others	Saccharides and Alcohols	5.87E+05	6.45E+05	6.51E+05	6.72E+05	--	--	cluster2
Phenethylamine	1.22E+02	1.05E+02	1.21E+02	C8H11N	[M+H]+	Others	Others	2.29E+06	1.02E+06	1.52E+07	1.14E+07	C05332	ko00360,ko01100	cluster3
Pyridoxine	1.70E+02	1.34E+02	1.69E+02	C8H11NO3	[M+H]+	Others	Vitamin	7.34E+05	4.28E+05	9.30E+05	1.07E+06	C00314	ko00750,ko01100	cluster3
N-Hydroxy tryptamine	1.77E+02	1.60E+02	1.76E+02	C10H12N2O	[M+H]+	Others	Others	8.89E+04	5.96E+04	2.81E+07	3.62E+07	C17203	ko00380,ko01100	cluster3
Inositol	1.79E+02	8.70E+01	1.80E+02	C6H12O6	[M-H]-	Others	Saccharides and Alcohols	8.34E+05	7.19E+05	2.34E+06	2.98E+06	C00137	ko00052,ko00053,ko00562,ko01100,ko02010,ko04070	cluster3
Cis-p-coumaric acid 4-O-glucoside	3.25E+02	1.63E+02	3.26E+02	C15H18O8	[M-H]-	Others	Others	3.54E+05	1.86E+05	7.38E+05	4.39E+05	--	--	cluster3
Vanillic acid-C-glucoside	3.29E+02	2.09E+02	3.30E+02	C14H18O9	[M-H]-	Others	Others	7.90E+04	6.54E+04	1.52E+06	1.62E+06	--	--	cluster3
D-(+)-Sucrose	3.41E+02	1.19E+02	3.42E+02	C12H22O11	[M-H]-	Others	Saccharides and Alcohols	7.77E+06	7.26E+06	1.48E+07	1.43E+07	C00089	ko00052,ko00500,ko01100,ko02010	cluster3
Isomaltulose	3.41E+02	8.90E+01	3.42E+02	C12H22O11	[M-H]-	Others	Saccharides and Alcohols	5.79E+06	5.76E+06	1.42E+07	1.33E+07	--	--	cluster3
Melibiose	3.41E+02	1.13E+02	3.42E+02	C12H22O11	[M-H]-	Others	Saccharides and Alcohols	1.24E+05	1.07E+05	2.51E+05	4.36E+05	C05402	ko00052,ko01100,ko02010	cluster3
Galactinol	3.41E+02	1.79E+02	3.42E+02	C12H22O11	[M-H]-	Others	Saccharides and Alcohols	8.97E+05	7.35E+05	1.87E+06	3.17E+06	--	--	cluster3
Trehalose 6-phosphate	4.21E+02	2.41E+02	4.22E+02	C12H23O14P	[M-H]-	Others	Saccharides and Alcohols	7.79E+04	9.00E+00	2.07E+05	1.64E+05	C00689	ko00500,ko01100	cluster3
4-C-β-D-Glucose-1,3,6-trihydroxy-7-methoxyxanthone	4.35E+02	3.45E+02	4.36E+02	C20H20O11	[M-H]-	Others	Xanthone	1.32E+04	5.95E+03	1.86E+05	1.45E+05	--	--	cluster3
Panose	5.03E+02	1.79E+02	5.04E+02	C18H32O16	[M-H]-	Others	Saccharides and Alcohols	1.40E+06	1.45E+06	1.84E+06	1.90E+06	C00713	--	cluster3
N-Acetyl-D-galactosamine	2.22E+02	8.41E+01	2.21E+02	C8H15NO6	[M+H]+	Others	Others	9.90E+04	1.14E+05	1.05E+05	1.87E+05	--	--	cluster4
N-Acetyl-β-D-mannosamine	2.22E+02	1.38E+02	2.21E+02	C8H15NO6	[M+H]+	Others	Others	1.56E+05	1.63E+05	1.63E+05	3.48E+05	--	--	cluster4
Byzantionoside A	3.71E+02	2.09E+02	3.70E+02	C19H30O7	[M+H]+	Others	Others	1.36E+04	9.12E+03	7.70E+03	1.78E+04	--	--	cluster4
Riboflavin	3.77E+02	2.43E+02	3.76E+02	C17H20N4O6	[M+H]+	Others	Vitamin	5.50E+04	5.47E+04	5.44E+04	6.86E+04	C00255	ko00740,ko01100,ko01110,ko02010	cluster4
Benzyl-β-D-glucopyranosyl (1-6)-β-D-glucopyranoside	4.31E+02	2.69E+02	4.32E+02	C19H28O11	[M-H]-	Others	Others	1.70E+04	7.85E+04	9.00E+00	1.65E+05	--	--	cluster4
1-Aminocyclopropane-1-carboxylic acid	1.02E+02	5.61E+01	1.01E+02	C4H7NO2	[M+H]+	Others	Others	3.06E+05	4.34E+05	2.11E+05	2.19E+05	C01234	ko00270,ko01100,ko01110	cluster5
Nicotinic acid	1.24E+02	7.80E+01	1.23E+02	C6H5NO2	[M+H]+	Others	Vitamin	1.09E+06	1.22E+06	8.70E+05	1.19E+06	C00253	ko00760,ko00960,ko01100,ko01110	cluster5
Sedoheptulose	2.09E+02	5.90E+01	2.10E+02	C7H14O7	[M-H]-	Others	Saccharides and Alcohols	1.74E+05	2.65E+05	7.50E+04	1.25E+05	C02076	ko00710,ko01100	cluster5
Glucarate O-Phosphoric acid	2.89E+02	9.70E+01	2.90E+02	C6H11PO11	[M-H]-	Others	Saccharides and Alcohols	1.12E+06	9.05E+06	4.07E+05	2.19E+06	--	--	cluster5
N-Oleoylethanolamine	3.26E+02	6.21E+01	3.25E+02	C20H39NO2	[M+H]+	Others	Others	4.27E+05	1.26E+06	3.11E+05	6.08E+05	C20792	--	cluster5
4'-O-β-D-Glucosyl-5-O-methylvisamminol	4.53E+02	2.91E+02	4.52E+02	C22H28O10	[M+H]+	Others	Others	1.26E+04	3.28E+04	1.72E+04	3.75E+04	--	--	cluster5
Propyl2-(trimethylammonio)ethyl phosphate	5.44E+02	4.85E+02	5.43E+02	C28H50NO7P	[M+H]+	Others	Others	1.26E+06	2.35E+06	3.31E+05	1.32E+06	--	--	cluster5
1,1-Kestotetraose	6.65E+02	4.85E+02	6.66E+02	C24H42O21	[M-H]-	Others	Saccharides and Alcohols	7.00E+06	9.60E+06	4.69E+06	6.17E+06	--	--	cluster5
Maltotetraose	6.65E+02	1.61E+02	6.66E+02	C24H42O21	[M-H]-	Others	Saccharides and Alcohols	7.34E+05	9.64E+05	4.70E+05	5.46E+05	C02052	--	cluster5
3-Carbamyl-1-methylpyridinium(1-Methylnicotinamide)	1.38E+02	9.50E+01	1.37E+02	C7H9N2O	[M+H]+	Others	Others	7.65E+04	4.83E+04	1.77E+04	3.17E+04	C02918	ko00760,ko01100	cluster6
L-Arabitol	1.51E+02	5.90E+01	1.52E+02	C5H12O5	[M-H]-	Others	Saccharides and Alcohols	7.79E+04	7.38E+04	5.89E+04	5.85E+04	C00532	ko00040,ko01100	cluster6
Z-6-Hydroxy-7-methoxydihydroligustilide	2.37E+02	1.49E+02	2.36E+02	C13H16O4	[M+H]+	Others	Others	2.78E+04	2.92E+04	2.39E+04	2.42E+04	--	--	cluster6
Diethanolamine	1.06E+02	8.80E+01	1.05E+02	C4H11NO2	[M+H]+	Others	Others	4.90E+04	4.87E+04	4.18E+04	1.63E+04	C06772	ko00564	cluster7
5,7-Dyhydroxy-1(3H)-isobenzofuran-one	1.65E+02	1.21E+02	1.66E+02	C8H6O4	[M-H]-	Others	Others	3.32E+04	3.43E+04	3.06E+04	2.00E+04	--	--	cluster7
4-Methyl-5-thiazoleethanol	1.44E+02	1.13E+02	1.43E+02	C6H9NOS	[M+H]+	Others	Others	2.93E+04	1.58E+04	1.50E+04	3.83E+03	C04294	ko00730,ko01100	cluster8
Ribitol	1.51E+02	7.10E+01	1.52E+02	C5H12O5	[M-H]-	Others	Saccharides and Alcohols	1.41E+05	1.22E+05	1.04E+05	9.54E+04	C00474	ko00040,ko00740,ko01100	cluster8
Xylitol	1.51E+02	5.90E+01	1.52E+02	C5H12O5	[M-H]-	Others	Saccharides and Alcohols	7.60E+04	5.52E+04	6.97E+04	5.36E+04	C00379	ko00040,ko01100,ko02010	cluster8
Octadecenoic amide	2.82E+02	2.47E+02	2.81E+02	C18H35NO	[M+H]+	Others	Others	5.89E+04	5.31E+04	3.30E+04	1.51E+04	--	--	cluster8
Pyridoxine-5'-O-glucoside	3.32E+02	1.52E+02	3.31E+02	C14H21NO8	[M+H]+	Others	Vitamin	2.20E+05	1.64E+05	1.54E+05	7.55E+04	C03996	--	cluster8
D-(-)-Arabinose	1.49E+02	5.90E+01	1.50E+02	C5H10O5	[M-H]-	Others	Saccharides and Alcohols	6.61E+04	5.55E+04	5.84E+04	5.93E+04	--	--	cluster9

D-Glucose	1.79E+02	5.90E+01	1.80E+02	C6H12O6	[M-H]-	Others	Saccharides and Alcohols	1.84E+05	9.49E+04	1.14E+05	9.49E+04	C00031	ko00010,ko00030,ko00052,ko00500,ko00520,ko00901,ko01100,ko01110,ko02010,ko04933	cluster9
Mannitol	1.81E+02	1.01E+02	1.82E+02	C6H14O6	[M-H]-	Others	Saccharides and Alcohols	2.41E+05	1.04E+05	8.30E+04	7.45E+04	C00392	ko00051,ko01100,ko02010	cluster9
Dulcitol	1.81E+02	1.01E+02	1.82E+02	C6H14O6	[M-H]-	Others	Saccharides and Alcohols	9.49E+05	4.02E+05	3.35E+05	2.96E+05	C01697	ko00052,ko01100	cluster9
D-Sorbitol	1.81E+02	7.10E+01	1.82E+02	C6H14O6	[M-H]-	Others	Saccharides and Alcohols	7.65E+05	3.16E+05	2.57E+05	2.29E+05	C00794	ko00051,ko00052,ko01100,ko02010	cluster9
Gluconic acid	1.95E+02	7.49E+01	1.96E+02	C6H12O7	[M-H]-	Others	Saccharides and Alcohols	1.60E+07	6.24E+06	4.05E+06	4.82E+06	C00257	ko00030,ko01100,ko01110,ko01200	cluster9
D-Pantothenic Acid	2.20E+02	2.02E+02	2.19E+02	C9H17NO5	[M+H]+	Others	Vitamin	3.26E+06	1.09E+06	7.62E+05	8.98E+05	C00864	ko00410,ko00770,ko01100,ko01110	cluster9
(R)-Pantetheine	2.77E+02	1.46E+02	2.78E+02	C11H22N2O4S	[M-H]-	Others	Others	7.77E+04	1.58E+04	3.43E+04	9.00E+00	C00831	ko00332,ko00770	cluster9
4-Methoxyglucobrassicin	4.77E+02	9.70E+01	4.78E+02	C17H22N2O10S2	[M-H]-	Others	Glucosinolates	2.36E+05	1.02E+05	8.22E+03	1.34E+04	C08423	--	cluster9
Benzaldehyde	1.07E+02	7.70E+01	1.06E+02	C7H6O	[M+H]+	Others	Others	5.02E+05	1.68E+05	8.68E+05	4.88E+05	C00261	ko01100	cluster10
D-Glucose 6-phosphate	2.59E+02	9.70E+01	2.60E+02	C6H13O9P	[M-H]-	Others	Saccharides and Alcohols	4.54E+04	9.00E+00	2.35E+04	1.22E+04	C00092	ko00500,ko00562,ko00998,ko01100	cluster10
Isochlorogenic acid A	5.15E+02	3.53E+02	5.16E+02	C25H24O12	[M-H]-	Phenolic acids	Phenolic acids	1.14E+04	2.18E+04	2.90E+05	3.90E+04	--	--	cluster1
3,4-Dicaffeoylquinic acid	5.15E+02	3.53E+02	5.16E+02	C25H24O12	[M-H]-	Phenolic acids	Phenolic acids	1.21E+04	2.26E+04	3.77E+05	4.85E+04	--	--	cluster1
Terephthalic acid	1.65E+02	1.21E+02	1.66E+02	C8H6O4	[M-H]-	Phenolic acids	Phenolic acids	8.93E+04	2.17E+05	1.81E+05	9.27E+04	C06337	ko01100	cluster2
p-coumaroylmalic acid	2.79E+02	1.63E+02	2.80E+02	C13H12O7	[M-H]-	Phenolic acids	Phenolic acids	9.00E+00	1.75E+04	3.12E+04	2.31E+04	--	--	cluster2
4-Hydroxybenzaldehyde	1.21E+02	9.20E+01	1.22E+02	C7H6O2	[M-H]-	Phenolic acids	Phenolic acids	2.89E+05	2.83E+05	4.99E+05	8.67E+05	C00633	ko01100	cluster3
4-Hydroxybenzoic acid	1.37E+02	9.30E+01	1.38E+02	C7H6O3	[M-H]-	Phenolic acids	Phenolic acids	1.33E+06	1.47E+06	1.61E+06	1.69E+06	C00156	ko00130,ko00790,ko01100,ko01110	cluster3
Tyrosol	1.37E+02	1.19E+02	1.38E+02	C8H10O2	[M-H]-	Phenolic acids	Phenolic acids	2.84E+04	2.56E+04	1.86E+05	1.39E+05	C06044	ko00350,ko01100	cluster3
2,4-Dihydroxy benzoic acid	1.53E+02	1.09E+02	1.54E+02	C7H6O4	[M-H]-	Phenolic acids	Phenolic acids	7.28E+04	4.01E+04	2.89E+05	2.73E+05	--	--	cluster3
Protocatechuic acid	1.53E+02	1.09E+02	1.54E+02	C7H6O4	[M-H]-	Phenolic acids	Phenolic acids	1.32E+05	4.41E+04	3.55E+05	3.06E+05	C00230	ko00400,ko01100,ko01110	cluster3
Trans-p-Hydroxycinnamic acid	1.65E+02	1.47E+02	1.64E+02	C9H8O3	[M+H]+	Phenolic acids	Phenolic acids	2.77E+04	9.55E+04	2.62E+05	2.31E+05	--	--	cluster3
p-Coumaric acid	1.65E+02	1.19E+02	1.64E+02	C9H8O3	[M+H]+	Phenolic acids	Phenolic acids	8.13E+04	2.68E+05	7.54E+05	6.97E+05	C00811	ko00130,ko00350,ko00940,ko00950,ko00999,ko01100,ko01110	cluster3
Methyl p-coumarate	1.79E+02	1.47E+02	1.78E+02	C10H10O3	[M+H]+	Phenolic acids	Phenolic acids	9.00E+00	9.00E+00	3.36E+04	2.55E+04	--	--	cluster3
Trans-4-Hydroxycinnamic Acid Methyl Ester	1.77E+02	1.45E+02	1.78E+02	C10H10O3	[M-H]-	Phenolic acids	Phenolic acids	9.00E+00	9.00E+00	9.55E+04	7.41E+04	--	--	cluster3
Caffeic acid	1.79E+02	1.35E+02	1.80E+02	C9H8O4	[M-H]-	Phenolic acids	Phenolic acids	9.00E+00	9.00E+00	2.14E+05	1.25E+05	C01197	ko00940,ko01100,ko01110	cluster3
Ferulic acid	1.93E+02	1.34E+02	1.94E+02	C10H10O4	[M-H]-	Phenolic acids	Phenolic acids	1.03E+05	5.74E+04	2.19E+06	8.32E+05	C01494	ko00940,ko01100,ko01110	cluster3
Salicylic acid O-glycoside	2.99E+02	1.37E+02	3.00E+02	C13H16O8	[M-H]-	Phenolic acids	Phenolic acids	9.00E+00	9.00E+00	1.05E+06	1.16E+06	--	--	cluster3
Isosalicylic acid O-glycoside	2.99E+02	1.37E+02	3.00E+02	C13H16O8	[M-H]-	Phenolic acids	Phenolic acids	9.00E+00	9.00E+00	6.76E+05	7.92E+05	--	--	cluster3
Glucosyloxybenzoic acid	2.99E+02	1.37E+02	3.00E+02	C13H16O8	[M-H]-	Phenolic acids	Phenolic acids	9.00E+00	9.00E+00	1.01E+06	1.18E+06	--	--	cluster3
2,5-Dihydroxy benzoic acid O-hexside	3.15E+02	1.53E+02	3.16E+02	C13H16O9	[M-H]-	Phenolic acids	Phenolic acids	8.98E+05	7.42E+05	8.36E+06	9.99E+06	--	--	cluster3
Cimidahurinine	3.15E+02	1.53E+02	3.16E+02	C14H20O8	[M-H]-	Phenolic acids	Phenolic acids	2.75E+04	1.76E+04	6.39E+05	5.37E+05	--	--	cluster3
5-(2-Hydroxyethyl)-2-O-glucosylohenol	3.15E+02	1.53E+02	3.16E+02	C14H20O8	[M-H]-	Phenolic acids	Phenolic acids	1.31E+04	1.96E+04	4.37E+05	3.65E+05	--	--	cluster3
1'-O-vanilloyl-β-D-glucoside	3.29E+02	1.67E+02	3.30E+02	C14H18O9	[M-H]-	Phenolic acids	Phenolic acids	6.78E+04	7.91E+04	4.98E+05	3.04E+05	--	--	cluster3
3,4,5-Trimethoxyphenyl-β-D-Glucopyranoside	3.29E+02	1.67E+02	3.30E+02	C15H22O8	[M-H]-	Phenolic acids	Phenolic acids	2.03E+06	1.33E+06	3.32E+06	5.26E+06	--	--	cluster3
1-O-Galloyl-β-D-glucose	3.31E+02	1.69E+02	3.32E+02	C13H16O10	[M-H]-	Phenolic acids	Phenolic acids	1.97E+06	4.45E+06	1.35E+07	2.53E+07	--	--	cluster3
6-O-Galloyl-β-D-glucopyranoside	3.33E+02	1.53E+02	3.32E+02	C13H16O10	[M+H]+	Phenolic acids	Phenolic acids	2.18E+04	6.58E+04	1.86E+05	3.56E+05	--	--	cluster3
3-O-(E)-p-Coumaroyl quinic acid	3.37E+02	1.91E+02	3.38E+02	C16H18O8	[M-H]-	Phenolic acids	Phenolic acids	3.48E+04	6.95E+04	1.32E+06	8.35E+05	--	--	cluster3
Coniferin	3.41E+02	1.79E+02	3.42E+02	C16H22O8	[M-H]-	Phenolic acids	Phenolic acids	1.39E+06	1.26E+06	1.46E+07	6.22E+06	C00761	ko00940	cluster3
Chlorogenic acid	3.53E+02	1.91E+02	3.54E+02	C16H18O9	[M-H]-	Phenolic acids	Phenolic acids	1.69E+04	2.06E+04	5.43E+05	1.90E+05	C00852	ko00940,ko00941,ko00945,ko01110	cluster3
Feruloyl glucose	3.55E+02	1.93E+02	3.56E+02	C16H20O9	[M-H]-	Phenolic acids	Phenolic acids	1.20E+06	6.25E+05	1.31E+07	7.06E+06	C17759	--	cluster3
Chlorogenic acid methyl ester	3.67E+02	1.91E+02	3.68E+02	C17H20O9	[M-H]-	Phenolic acids	Phenolic acids	9.00E+00	9.00E+00	2.15E+04	2.00E+04	--	--	cluster3
Syringin	3.71E+02	2.09E+02	3.72E+02	C17H24O9	[M-H]-	Phenolic acids	Phenolic acids	3.24E+05	2.58E+05	4.22E+05	5.60E+05	C01533	ko00940	cluster3
Sinapic acid-glycoside	3.85E+02	2.23E+02	3.86E+02	C17H22O10	[M-H]-	Phenolic acids	Phenolic acids	1.09E+07	1.00E+07	1.39E+07	1.24E+07	--	--	cluster3
1'-O-β-D-(3,4-Dihydroxyphenethyl)-O-caffeoyl-glucoside	4.77E+02	1.79E+02	4.78E+02	C23H26O11	[M-H]-	Phenolic acids	Phenolic acids	9.00E+00	9.00E+00	8.71E+05	7.05E+05	--	--	cluster3
3,5-Di-O-galloylshikimic acid	4.77E+02	1.69E+02	4.78E+02	C23H26O11	[M-H]-	Phenolic acids	Phenolic acids	9.00E+00	9.00E+00	6.96E+04	1.34E+05	--	--	cluster3
Benzoic acid	1.23E+02	7.90E+01	1.22E+02	C7H6O2	[M+H]+	Phenolic acids	Phenolic acids	2.38E+04	2.01E+04	1.57E+04	3.16E+04	C00180	ko00360,ko01100,ko01110	cluster4
1-(4-Methoxyphenyl)-1-propanol	1.65E+02	1.49E+02	1.66E+02	C10H14O2	[M-H]-	Phenolic acids	Phenolic acids	1.57E+04	1.62E+04	1.46E+04	1.84E+04	--	--	cluster4
5-Hydroxymethylfurfural	1.27E+02	9.70E+01	1.26E+02	C6H6O3	[M+H]+	Phenolic acids	Phenolic acids	1.21E+06	1.61E+06	9.68E+05	1.42E+06	C11101	--	cluster5
3,4-Dihydroxybenzaldehyde	1.37E+02	9.30E+01	1.38E+02	C7H6O3	[M-H]-	Phenolic acids	Phenolic acids	1.55E+06	1.74E+06	1.63E+06	1.73E+06	--	--	cluster5

Cinnamic acid	1.47E+02	7.70E+01	1.48E+02	C9H8O2	[M-H]-	Phenolic acids	Phenolic acids	7.52E+03	2.86E+04	2.72E+03	5.40E+03	C00423	ko00130,ko00360,ko00940,ko00999,ko01100,ko01110	cluster5
Sibiricose A6	5.47E+02	2.05E+02	5.48E+02	C23H32O15	[M-H]-	Phenolic acids	Phenolic acids	1.35E+04	1.74E+05	9.00E+00	9.00E+00	--	--	cluster5
4-Aminobenzoic acid	1.38E+02	1.20E+02	1.37E+02	C7H7NO2	[M+H]+	Phenolic acids	Phenolic acids	2.07E+05	1.64E+05	7.48E+04	9.11E+04	C00568	ko00790,ko01100	cluster6
Phthalic anhydride	1.49E+02	6.50E+01	1.48E+02	C8H4O3	[M+H]+	Phenolic acids	Phenolic acids	2.64E+05	2.80E+05	2.12E+05	2.34E+05	--	--	cluster6
4-MethoxycinnaMaldehyde	1.63E+02	9.11E+01	1.62E+02	C10H10O2	[M+H]+	Phenolic acids	Phenolic acids	4.16E+04	3.58E+04	2.74E+04	3.25E+04	--	--	cluster6
Glucosyringic Acid	3.59E+02	1.82E+02	3.60E+02	C15H20O10	[M-H]-	Phenolic acids	Phenolic acids	2.54E+05	3.41E+05	1.34E+04	3.63E+04	--	--	cluster6
Cinncassiol C1	3.81E+02	3.37E+02	3.82E+02	C20H30O7	[M-H]-	Phenolic acids	Phenolic acids	1.92E+05	2.82E+05	6.62E+03	9.35E+03	C17643	--	cluster6
Methyl-(2,4-dihydroxyphenyl)acetate	1.81E+02	1.35E+02	1.82E+02	C9H10O4	[M-H]-	Phenolic acids	Phenolic acids	2.96E+05	4.06E+04	1.24E+05	1.65E+05	--	--	cluster9
p-Hydroxyphenyl acetic acid	1.51E+02	1.07E+02	1.52E+02	C8H8O3	[M-H]-	Phenolic acids	Phenolic acids	4.83E+04	9.00E+00	6.51E+04	5.04E+04	C00642	ko00350,ko00360,ko01100	cluster10
3-(4-Hydroxyphenyl)-propionic acid	1.65E+02	1.19E+02	1.66E+02	C9H10O3	[M-H]-	Phenolic acids	Phenolic acids	5.09E+05	1.54E+05	4.45E+05	6.18E+05	C01744	--	cluster10
Sinapic acid	2.23E+02	1.93E+02	2.24E+02	C11H12O5	[M-H]-	Phenolic acids	Phenolic acids	2.26E+05	8.44E+04	3.10E+05	1.43E+05	C00482	ko00940,ko01100,ko01110	cluster10
3-Hydroxy-4-isopropylbenzylalcohol 3-glucoside	3.27E+02	1.65E+02	3.28E+02	C16H24O7	[M-H]-	Phenolic acids	Phenolic acids	3.52E+04	1.26E+04	2.54E+04	3.72E+04	--	--	cluster10
Aloeemodin-8-O-D-glucopyranoside	4.31E+02	2.69E+02	4.32E+02	C21H20O10	[M-H]-	Quinones	Anthraquinone	9.00E+00	9.00E+00	2.19E+05	1.88E+05	--	--	cluster3
Rhein-8-O-D-glucopyranoside	4.45E+02	2.83E+02	4.46E+02	C21H18O11	[M-H]-	Quinones	Anthraquinone	9.00E+00	1.27E+04	2.01E+05	3.46E+05	--	--	cluster3
Hydroxyaloe-emodin-O-glucoside	4.47E+02	2.85E+02	4.48E+02	C21H20O11	[M-H]-	Quinones	Anthraquinone	5.74E+04	1.33E+05	7.41E+06	1.24E+07	--	--	cluster3
Phosphoenolpyruvate	2.71E+02	2.25E+02	2.70E+02	C15H10O5	[M+H]+	Quinones	Quinones	7.13E+03	6.76E+03	9.00E+00	9.00E+00	C10294	--	cluster6
2-O-Galloyl-β-D-glucose	3.31E+02	1.69E+02	3.32E+02	C13H16O10	[M-H]-	Tannins	Tannin	1.84E+06	4.44E+06	1.44E+07	2.63E+07	--	--	cluster3
Ursolic acid	4.55E+02	4.55E+02	4.56E+02	C30H48O3	[M-H]-	Terpenoids	Triterpene	9.00E+00	9.00E+00	7.69E+04	8.57E+03	C08988	--	cluster1
Aucubin	3.45E+02	1.83E+02	3.46E+02	C15H22O9	[M-H]-	Terpenoids	Sesquiterpenoids	4.36E+04	7.13E+04	5.36E+05	6.47E+05	C09771	--	cluster3
Maslinic acid	4.71E+02	4.71E+02	4.72E+02	C30H48O4	[M-H]-	Terpenoids	Triterpene	1.70E+04	1.52E+05	9.00E+00	5.96E+03	C16939	--	cluster5
